# MXene-Based Electrocatalysts for Water Splitting: Material Design, Surface Modulation, and Catalytic Performance

**DOI:** 10.3390/ijms26168019

**Published:** 2025-08-19

**Authors:** Mohammad R. Thalji, Farzaneh Mahmoudi, Leonidas G. Bachas, Chinho Park

**Affiliations:** 1 KENTECH Institute for Hydrogen Energy, Korea Institute of Energy Technology (KENTECH), 21 KENTECH-gil, Naju 58330, Jeollanam-do, Republic of Korea; 2 Department of Chemistry, University of Miami, Coral Gables, FL 33146, USA; fxm676@miami.edu (F.M.); bachas@miami.edu (L.G.B.)

**Keywords:** MXene, electrocatalysis, water splitting, hydrogen evolution reaction (HER), oxygen evolution reaction (OER), composite materials, structural engineering

## Abstract

Developing efficient and sustainable hydrogen production technologies is critical for advancing the global clean energy transition. This review highlights recent progress in the design, synthesis, and electrocatalytic applications of MXene-based materials for electrochemical water splitting. It discusses the fundamental mechanisms of the hydrogen evolution reaction (HER) and oxygen evolution reaction (OER), and the structure–function relationships that govern electrocatalytic behavior. Emphasis is placed on the intrinsic structural and surface properties of MXenes, such as their layered architecture and tunable surface chemistry, which render them promising candidates for electrocatalysis. Despite these advantages, several practical limitations hinder their full potential, including oxidation susceptibility, restacking, and a limited number of active sites. Several studies have addressed these challenges using diverse engineering strategies, such as heteroatom doping; surface functionalization; and constructing MXene-based composites with metal chalcogenides, oxides, phosphides, and conductive polymers. These modifications have significantly improved catalytic activity, charge transfer kinetics, and long-term operational stability under various electrochemical conditions. Finally, this review outlines key knowledge gaps and emerging research directions, including defect engineering, single-atom integration, and system-level design, to accelerate the development of MXene-based electrocatalysts for sustainable hydrogen production.

## 1. Introduction

The escalating global demand for energy, driven by population growth and industrial expansion, has intensified the urgent need for sustainable solutions amid rising environmental concerns and the depletion of fossil fuel reserves [1]. Although primary renewable energy sources, such as solar, wind, and tidal power, offer clean electricity generation [2], their inherent intermittency and geographic dependence present challenges to widespread deployment across all sectors. Unlike conventional fuels, hydrogen is a versatile energy carrier, facilitating storage, transport, and utilization of renewable energy. Its unique properties [3] make hydrogen especially well-suited for decarbonizing sectors [4] where direct electrification remains impractical or inefficient, including steel manufacturing, aviation, maritime shipping, and long-haul transportation. In this context, electrochemical hydrogen production via water splitting has garnered significant attention as a scalable, carbon-free approach, enabling green hydrogen generation using renewable electricity and eliminating reliance on fossil-based feedstocks [5,6].

Electrocatalytic water splitting comprises two interrelated half-reactions: the oxygen evolution reaction (OER) at the anode and the hydrogen evolution reaction (HER) at the cathode. The thermodynamic potential required to drive these processes under standard conditions is 1.23 V vs. the reversible hydrogen electrode (RHE). However, OER and HER are inherently sluggish, necessitating substantial overpotentials to overcome activation barriers and achieve practical reaction rates [7,8,9]. Consequently, highly efficient electrocatalysts are essential for accelerating these reactions and reducing energy consumption. Although noble metals, such as iridium/ruthenium (Ir/Ru) oxides for OER and platinum (Pt) for HER, exhibit outstanding catalytic performance, their high costs and limited natural abundance hinder large-scale application [8]. To address these challenges, intensive research efforts have focused on developing cost-effective and earth-abundant catalytic materials that support the advancement of sustainable hydrogen production technologies [7,8,9,10]. An effective electrocatalyst must possess excellent intrinsic activity, a high accessible surface area, and robust operational stability. Achieving these qualities requires rational design strategies that enable rapid electron transfer and efficient gas release, thereby minimizing overpotentials while sustaining high current densities [11]. A range of nanomaterials, including metal–organic frameworks (MOFs) [12,13], metal carbides [14,15], phosphides [16,17], sulfides [18,19], nitrides [20], oxides [21,22], and their composite forms [2,23,24,25], have been developed as water-splitting electrocatalysts. In addition, two-dimensional (2D) layered materials, such as graphitic carbon nitride, layered double hydroxides (LDHs), and graphene, have attracted increasing attention, owing to their tunable properties and high surface areas [5,26]. Carbon materials are often employed as conductive supports to stabilize transition metal nanostructures, improving catalyst dispersion and electron conductivity. However, these hybrids can experience catalyst agglomeration or structural instability during prolonged operation at high current densities.

In response to these limitations, early transition metal carbides and/or nitrides (MXenes) have emerged as attractive water-splitting electrocatalysts [27]. MXenes feature tunable layered structures, excellent electrical conductivity, high mechanical strength, pronounced hydrophilicity, and large electroactive surface areas with modifiable surface terminations [2,5,11,27,28]. Their catalytic activity can be further enhanced through surface modification, defect engineering, heteroatom incorporation, or integration with other active materials, collectively increasing the density and accessibility of catalytic sites [2,29]. MXene-based materials have been widely investigated for various energy applications, including batteries [30,31], supercapacitors [32,33], and electrocatalytic systems [29,34,35]. Their heterostructures, characterized by strong interfacial interactions, inhibit restacking and aggregation of MXene layers, mitigate surface oxidation, and improve electrical conductivity and stability [3,5,18]. These features stem from enhanced electron transfer via interfacial bonding; optimized adsorption energies for reaction intermediates, often modulated by lattice strain; and interface-induced charge redistribution [5,18]. Several reviews have documented MXene synthesis methods, structural characteristics, and energy-related functionalities [2,36,37,38], reaffirming their relevance to next-generation energy storage and conversion.

Molecular-level modulation strategies, detailed structure–property–performance relationships, and the integration of MXene-derived catalysts into practical devices are critically important for the realization of practical hydrogen generation systems. This review uniquely addresses these emerging frontiers by thoroughly analyzing synthetic pathways, defect engineering, heteroatom doping, and hybrid architecture. Our perspective aims to complement and expand upon the prior literature, bridging fundamental insights with application-driven design strategies. Furthermore, most published review articles reported applications of MXene-based composites in different fields. This review focuses on recently published research on MXene-based composites used for electrocatalytic HER and OER in water splitting. It presents an in-depth assessment of recent progress in designing and optimizing MXene-based electrocatalysts, focusing on their applications in water splitting. Specifically, Section 2 covers the fundamentals of water-splitting reactions, laying the groundwork for further discussion. Section 3 examines synthesis methods; structural features; key physicochemical properties of MXenes; and strategies for surface engineering, defect modulation, and interface design. Section 4 highlights recent advances in compositional and architectural design approaches for enhancing the electrocatalytic activity and operational durability of MXene-derived composite materials. Finally, Section 5 discusses prevailing challenges, emerging opportunities, and future research directions. This review aims to provide a valuable resource for researchers dedicated to developing advanced MXene-based electrocatalysts for efficient and sustainable water splitting.

## 2. Fundamentals of Water Splitting

Water splitting is a cornerstone for sustainable hydrogen production, enabling water’s electrochemical conversion into molecular hydrogen (H_2_) and oxygen (O_2_) [3,39]. This process comprises two kinetically distinct half-reactions: HER and OER [40,41]. Each reaction requires tailored electrocatalysts to facilitate charge transfer and promote favorable reaction kinetics. Although the theoretical minimum cell voltage for overall water splitting is 1.23 V vs. RHE [6] under standard conditions, practical systems typically demand higher applied potentials to overcome significant kinetic energy barriers, particularly within the multi-step OER pathway. Electrocatalysts play a critical role; they reduce activation energy thresholds and accelerate reaction rates, thereby improving energy efficiency [8,42]. The HER mechanism involves reducing protons to H_2_, which requires electrocatalysts with optimal hydrogen binding energies to enable effective adsorption and recombination of intermediates. In contrast, OER is more complex, proceeding via coupled proton–electron transfer steps and the formation of unstable, high-energy surface intermediates. This complexity renders OER the rate-limiting step in overall water electrolysis [43]. MXene materials have garnered significant attention as next-generation electrocatalysts [8,44] owing to their intrinsic metallic conductivity, chemically tunable surfaces, and high specific surface areas. The most important factors affecting their electrocatalytic activity are the overpotential, Tafel slope, exchange current density, operational durability, energy requirement parameters, mechanistic pathways, and catalyst robustness. Precise control over surface chemistry and defect engineering allows tuning the adsorption energies of reaction intermediates and enhancing catalytic kinetics [2,45]. An in-depth understanding of these physical and chemical parameters is crucial for designing efficient electrocatalysts for green hydrogen production.

### 2.1. Thermodynamics and Kinetics of the HER and OER

Complex thermodynamic and kinetic processes govern the HER and OER. In water splitting, HER occurs at the cathode (via proton or water reduction), whereas OER occurs at the anode (via water oxidation). Although the overall reactions are simple, both involve multiple steps and adsorbed intermediates, especially for OER. The OER is inherently sluggish due to its complicated, multi-step mechanism involving a concerted four-electron transfer process and the sequential formation of intermediates, such as *OH, *O, and *OOH. Each intermediate requires a distinct adsorption/desorption step and electron transfer event. This sequence significantly raises the reaction energy barrier, resulting in high overpotentials and slow kinetics, particularly under alkaline conditions where proton availability is limited [41]. Additionally, the formation and cleavage of strong O–H and O–O bonds demand considerable energy, posing further challenges in catalyst design. By contrast, HER proceeds through a simpler two-electron mechanism, though its kinetics are still highly pH-dependent due to variations in proton availability and activation barriers. HER primarily follows the Volmer–Heyrovsky or Volmer–Tafel mechanism in acidic media. In the Volmer step, a proton is electrochemically adsorbed onto the metal surface to form a metal–hydrogen intermediate (*M*-H*), which then undergoes either electrochemical desorption (Heyrovsky step) or chemical recombination (Tafel step) to release H_2_ gas [7,46]:

Acidic medium HER:

$$M+H^{+}+e^{-}\to M-H^{*}\;(\mathrm{Volmer})$$

$$M-H^{*}+H^{+}+e^{-}\to M+H_{2}\;(\mathrm{Heyrovsky})$$

$$2M-H^{*}\to {2M+H}_{2}\;(\mathrm{Tafel})$$

Here, the availability of solvated protons (H^+^) favors effective hydrogen adsorption and H_2_ evolution. The rate-determining step depends on the hydrogen binding energy to the catalyst surface. In alkaline media, the HER is more challenging because water molecules, instead of free protons, serve as the proton source; the initial water dissociation (Volmer step) thus becomes rate-determining, requiring higher activation energy and introducing a further kinetic barrier.

Alkaline medium HER:

$$M+H_{2}O+e^{-}\to OH^{-}+M-H^{*}\;(\mathrm{Volmer})$$

$$M-H^{*}+H_{2}O+e^{-}\to M+{OH^{-}+H}_{2}\;(\mathrm{Heyrovsky})$$

$$2M-H^{*}\to {2M+H}_{2}\;(\mathrm{Tafel})$$

M represents a metal atom on the catalyst surface, and *M*–H* is an intermediate that presents the adsorbed H on the metal atom (M). An efficient alkaline HER catalyst must display a Gibbs free energy for hydrogen adsorption (ΔG_H*_) near zero [47] and promote efficient water dissociation [48]. For example, incorporating oxophilic transition metals or constructing heterostructures with hydrophilic sites can enhance water cleavage and accelerate the Volmer step, thus improving overall HER kinetics under alkaline conditions [49].

For OER, the mechanism typically starts with the adsorption of a water molecule or hydroxide ion at the active site (*M*), followed by stepwise deprotonation and oxidation to form *OH, *O, and *OOH intermediates, culminating in O_2_ evolution [5]. The stabilization of these intermediates is highly dependent on the catalyst’s electronic structure, which influences the energetics of each reaction pathway. The complete four-electron transfer pathway increases reaction complexity and leads to substantial energy losses from kinetic bottlenecks [7,46].

Acidic medium OER:

$$H_{2}O+M\to M-OH^{*}+H^{+}+e^{-}$$

$$M-OH^{*}\to M-O^{*}+H^{+}+e^{-}$$

$$M-O^{*}+H_{2}O\to M-OOH^{*}+H^{+}+e^{-}$$

$$M-OOH^{*}\to O_{2}+H^{+}+e^{-}+M$$

Alkaline medium OER:

$$OH^{-}+M\to M-OH^{*}+e^{-}$$

$$M-OH^{*}+OH^{-}\to M-O^{*}+H_{2}O+e^{-}$$

$$M-O^{*}+OH^{-}\to M-OOH^{*}+e^{-}$$

$$M-OOH^{*}+OH^{-}\to O_{2}+H_{2}O+e^{-}+M$$

During OER, catalysts may undergo in situ surface reconstruction to form active oxyhydroxide or oxide phases, especially under highly alkaline or acidic conditions. High-valence transition metal sites often constitute the principal active centers.

The inherent differences in complexity and kinetic barriers between the HER and OER present critical challenges for catalyst design. The OER, which involves a four-electron transfer process with multiple reaction intermediates, demands catalysts that can effectively stabilize and activate various oxygen-containing species while minimizing energy losses. In contrast, HER mechanisms depend strongly on the electrolyte environment: efficient catalysts must facilitate proton reduction in acidic media or promote water dissociation under alkaline conditions. Addressing these distinct mechanistic requirements is pivotal for rationalizing efficient bifunctional electrocatalysts for overall water splitting.

### 2.2. Electrocatalytic Performance Metrics

Electrocatalysts for water splitting are evaluated using standardized performance indicators reflecting intrinsic activity and long-term durability [1,2]. These parameters help elucidate HER and OER mechanisms, enable benchmarking across different materials and studies, and inform strategies for rational catalyst design. One of the primary indicators of electrocatalytic performance is the overpotential, which quantifies the extra voltage required to drive the reaction beyond its thermodynamic threshold. Lower overpotentials indicate higher catalytic efficiency. MXene-based systems have substantially reduced overpotential facilitated by their tunable surface chemistry, adjustable interlayer spacing, and compositional versatility, which are enabled by heteroatom doping and hybridization. Another critical metric is the Tafel slope, which relates the overpotential to the logarithm of the current density, providing insights into reaction kinetics [50]. A smaller Tafel slope generally denotes faster kinetics and more efficient charge transfer. MXene-based catalysts often exhibit reduced Tafel slopes enabled by structural modifications, including defect engineering, heterostructure construction, and atomic-scale doping, each contributing to increased density and accessibility of active sites.

The exchange current density (j_o_) directly measures the intrinsic electron transfer rate at the equilibrium [51]. High values of j_o_ indicate a catalyst’s ability to initiate the reaction with minimal driving force [52]. In MXenes, this parameter is highly sensitive to the nature of surface termination groups, which govern local electronic structure and intermediate adsorption energetics [53]. Incorporating conductive additives or transition metals can result in further improvements by boosting electron mobility and interfacial reactivity. Turnover frequency (TOF) directly measures site-specific catalytic efficiency by quantifying the number of reactant molecules converted per active site per unit time [54]. TOF is especially valuable for comparing catalysts with different surface areas and morphologies. Accurate TOF calculation in MXene-based systems requires precise determination of the number of electrochemically active sites, often determined by chemisorption analysis or electrochemical surface area (E_CSA_) measurements [55]. Beyond activity, electrocatalysts’ long-term durability and structural stability under operational conditions are paramount for practical applications. Robustness is typically evaluated via chronoamperometry (CA), chronopotentiometry (CP), and cyclic voltammetry (CV) over extended timescales or repeated cycles, which reveals catalyst stability and resistance to degradation. Table 1 summarizes the key electrocatalytic performance metrics, corresponding characterization techniques, and MXene-specific design strategies for optimizing HER and OER activity.

Post-catalysis characterization using a comprehensive suite of structural and electrochemical techniques, including X-ray diffraction (XRD), X-ray photoelectron spectroscopy (XPS), transmission electron microscopy (TEM), high-resolution TEM (HR-TEM), Raman spectroscopy, and electrochemical impedance spectroscopy (EIS), provides critical insights into degradation pathways [56], such as surface oxidation, phase transformation, and loss of crystallinity or conductivity. These diagnostic approaches enable the identification of performance-loss mechanisms and inform strategies to enhance catalyst resilience. Notably, MXene-based electrocatalysts have exhibited promising structural and electrochemical stability during HER and OER [56,57] due to their layered morphology, high electrical conductivity, and chemically tunable surfaces. Table 2 summarizes the principal post-catalysis characterization techniques utilized for MXene-based HER/OER systems, outlining their roles in evaluating catalyst integrity, surface chemistry, and operational durability.

### 2.3. Role of Electrocatalyst Surface and Interface in Reaction Pathways

The catalytic activity of MXenes in water-splitting reactions is strongly governed by their surface and interfacial characteristics, which dictate adsorption energetics, charge redistribution, and overall reaction kinetics. While bulk properties such as electrical conductivity and mechanical robustness are essential, the surface and interface design ultimately dictate electrocatalytic performance [7]. MXenes provide an exceptionally versatile platform for surface engineering due to their 2D morphology, abundant and tunable surface terminations (e.g., –OH, –O, –F), and high intrinsic conductivity. These features facilitate fine-tuning of the electronic structure at the atomic level. Achieving an optimal ΔG_H*_ is essential for the HER to satisfy the Sabatier principle [57]. Weak hydrogen adsorption produces poor surface coverage, whereas firm binding impedes H_2_ release. By modulating surface terminations or incorporating heteroatoms (e.g., N, S, P), MXenes can approach the ideal ΔG_H*_, enhancing HER kinetics.

In contrast, the OER presents more complex catalytic requirements, involving multi-step electron transfers and stabilizing various reaction intermediates (e.g., *OH, *O, *OOH) [58]. Strategies such as defect engineering, lattice strain modulation, and hybridization with catalytically active phases (e.g., metal oxides or phosphides) have been shown to optimally adjust the binding energies of these intermediates, reduce activation barriers, and facilitate oxygen desorption [59]. Furthermore, interface engineering is critical for maximizing the electrocatalytic performance of MXenes. The construction of heterostructures can create synergistic effects by integrating MXenes with other functional materials such as transition metal phosphides, sulfides, or oxides. These include generating new active sites at the interface, enhancing charge separation, and developing interfacial electric fields that guide electron flow. Such configurations may also induce localized modulation in the density of states near the Fermi level, promoting higher activity and improved selectivity. Additionally, advanced MXene-based architectures with spatially separated HER and OER active sites within a single framework can suppress the cross-recombination of H_2_ and O_2_, further boosting the overall efficiency of water-splitting systems.

## 3. MXene: Synthesis, Structures, and Properties

### 3.1. Synthetic Strategies of MXenes

**Figure 1:**
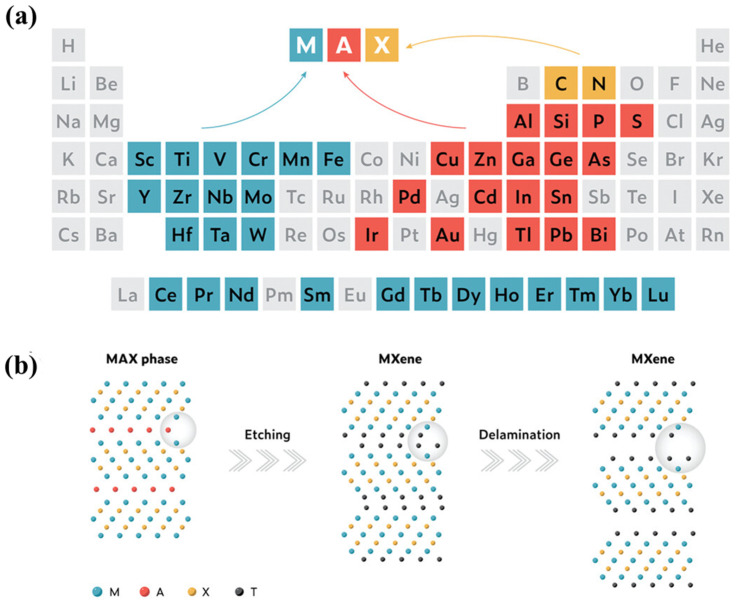
(**a**) Periodic table highlighting the elements used in the structure of MAX phases; (**b**) schematic illustration of the synthesis of layered M_3_X_2_T*_x_* MXene by the selective etching of the “A” element from a M_3_AX_2_ MAX phase. Reproduced with permission from [66].

Since the first discovery of 2D MXene by Gogotsi and colleagues in 2011 [60,61], these materials have attracted significant interest for their excellent electrical conductivity, rich surface chemistry, exceptional mechanical strength, and distinctive hydrophilicity [62,63]. MXenes possess a general formula of M_n+1_X_n_T*_x_*, where ‘M’ denotes an early transition metal (e.g., Ti, V, Mo, Ta), ‘X’ refers to nitrogen and/or carbon (Figure 1), and ‘T’ represents surface functional moieties (–OH, –F, –O). In the parent MAX phase, ‘A’ is usually a group IIIA or IVA element (e.g., Al, Si, Ga) [64]. The first synthesis of Ti_3_C_2_ MXene was accomplished through selective etching of the A-site element, Al, from Ti_3_AlC_2_ (the MAX phase precursor) using hydrofluoric acid (HF). This method remains widely adopted due to its simplicity, efficiency, and adaptability to various MAX precursors [65]. The resulting etching process removes the Al atomic layers, yielding stacked Ti_3_C_2_ layers with surface terminations introduced during etching, such as –F, –OH, and –O. These surface groups play a crucial role in governing the physical and chemical properties of the resulting MXene.

**Figure 2:**
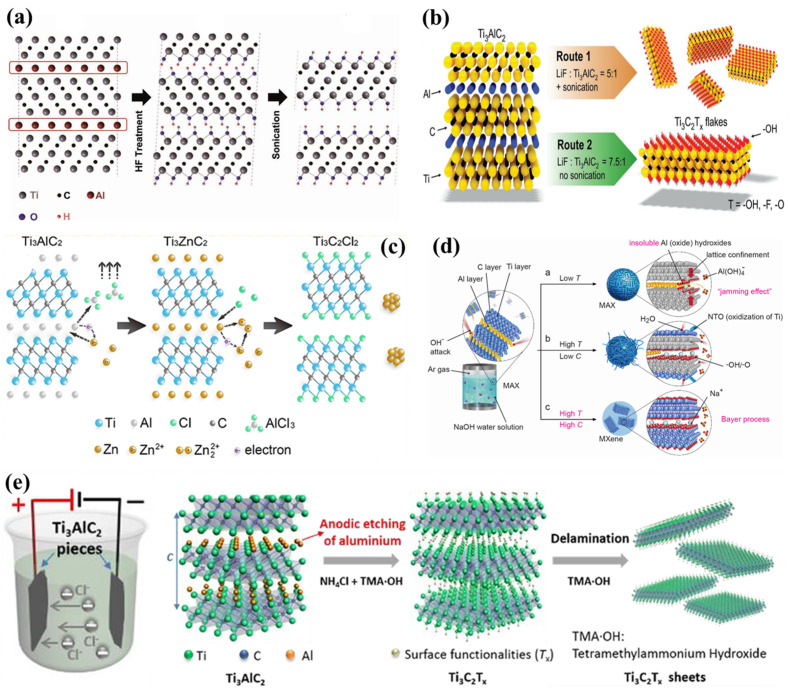
(**a**) Schematic illustration of Ti_3_AlC_2_ exfoliation using HF treatment followed by ultrasonic delamination in methanol to isolate Ti_3_C_2_T*_x_* layers (reproduced with permission from [62]); (**b**) synthesis of Ti_3_C_2_T*_x_* flakes using LiF and HCl by two routes (reproduced with permission from [69]); (**c**) synthesis of Ti_3_C_2_Cl_2_ with molten salt etching, where mixtures of Ti_3_AlC_2_ and ZnCl_2_ in ratios ranging from 1:1 to 1:6 were thermally treated at 550 °C for durations between 0.5 and 5 h (reproduced with permission from [70]); (**d**) alkaline etching strategy using aqueous NaOH solution under controlled temperature and concentration conditions to obtain Ti_3_AlC_2_ (reproduced with permission from [71]); and (**e**) configuration of an electrochemical cell with anodic etching of bulk Ti_3_AlC_2_ in a binary aqueous electrolyte system (reproduced with permission from [72]).

Various etching routes have emerged to remove the A-layer selectively from MAX phases, including direct treatment with HF, in situ HF generation from fluoride salts and HCl [60], alkali-assisted approaches [67], molten salt methods [6], mechanical ball milling [68], and electrochemical etching [36]. The choice of etching technique critically influences the resulting structure of the MXene, its surface terminations, and overall electrical conductivity [55]. HF etching remains the earliest [62] and most widely utilized approach (Figure 2a). This process relies on the high reactivity of fluoride ions with the A-site (commonly Al), facilitating selective removal while preserving the M–X framework and introducing –O, –OH, and –F surface groups [36]. However, despite its effectiveness, HF poses significant safety concerns due to its corrosivity and toxicity, motivating the search for safer alternatives. One such advancement was reported by Ghidiu et al. [60], who demonstrated a milder etching method based on the use of fluoride salts and HCl (Figure 2b). This method generates HF in situ, reducing direct exposure and associated handling risks while achieving comparable delamination and functionalization outcomes.

Another emerging route for MXene synthesis is molten salt etching (Figure 2c), which removes Al from the Ti_3_AlC_2_ MAX phase under high-temperature, inert conditions without hazardous chemicals [6]. This method avoids corrosive acids but requires elevated temperatures and poses safety considerations related to thermal management. The resulting MXene typically retains the characteristic accordion-like morphology of the layered structure. Mechanical ball milling has also gained attention as a solvent-free, scalable approach to synthesize MXenes featuring hierarchical porosity and enhanced surface area [1]. Xue et al. [68] reported a fluorine-free ball-milling strategy that combines tetramethylammonium hydroxide (TMAOH) and lithium chloride to delaminate Ti_3_C_2_ MXene. This method relies on mechanically driven etching and exfoliation mechanisms, offering an environmentally friendly alternative to conventional chemical etching. Alkali-assisted etching methods utilize concentrated alkaline solutions to selectively dissolve the Al layers (Figure 2d), yielding MXenes with fluorine-free surfaces and distinctive termination groups [67]. For example, Li et al. [71] synthesized high-purity Ti_3_C_2_ MXene (92 wt. %) by treatment with 27.5 M NaOH at 270 °C, obtaining fluorine-free MXenes with predominantly –O- and –OH-terminated surfaces. Deep eutectic solvents (DES) have recently facilitated anhydrous ionic thermal synthesis routes. Wu et al. [73] demonstrated that DES enables the production of phase-pure Ti_3_C_2_ MXene by leveraging its low vapor pressure and strong solvation capacity, providing an alternative medium for high-quality MXene fabrication.

Electrochemical etching represents another advanced method, enabling spatially and temporally controlled removal of the A-layer atoms using programmed voltage profiles during cyclic voltammetry (Figure 2e). This method offers advantages such as selective etching, scalable film formation, and tunable surface chemistry [2,36]. However, the choice of electrolyte is critical; anodic etching in conventional media like NaCl, HCl, or HF can cause unwanted dissolution of both Ti and Al, leading to amorphous carbon byproducts [74]. To overcome these challenges, Wong et al. [75] developed a simple and efficient electrochemical route using a mixed LiOH/LiCl electrolyte, producing fluorine-free Ti_3_C_2_T*_x_* MXene terminated mainly with –Cl groups and attaining a high etching efficiency of 92.2%. These diverse etching strategies expand the synthetic toolbox for tailoring MXene composition, structure, and surface chemistry, enabling rational design for targeted electrocatalytic applications and other advanced technologies.

### 3.2. Physical Properties and Structural Engineering of MXene-Based Materials

MXenes constitute a versatile class of layered 2D materials distinguished by exceptional conductivity, tunable optical, and robust mechanical properties [76]. Among these, Ti_3_C_2_T*_x_* is the most extensively studied MXene, exhibiting electrical conductivity ranging from 850 to 9880 S cm^−1^ [76]. Such variation in conductivity primarily arises from differences in synthesis conditions, including defect density, degree of surface functionalization, extent of delamination, interlayer spacing, and lateral flake dimensions [77]. Generally, lower HF concentrations and shorter etching durations lead to MXenes with fewer defects and larger lateral sizes, thereby enhancing conductivity. Additionally, surface terminations (–F, –OH, –O) and intercalated species significantly modulate electronic behavior by affecting band structure, Fermi level position, and carrier concentration [66]. Both –F and –OH functional groups have been reported to facilitate improved charge transport and contribute positively to electrical conductivity [78]. In addition to their electrical properties, MXenes possess high thermal conductivity due to their layered structure and the metallic nature of the transition metal in their lattice. This thermal behavior makes them attractive for applications in thermal management, such as catalysis, energy storage, and heat dissipation technologies operating under elevated temperatures [3]. The inherent thermal stability of MXenes further supports their utilization in harsh operating environments.

MXenes also exhibit remarkable linear and nonlinear optical properties stemming from unique band structures that may feature direct or indirect band gaps and topological characteristics [79,80,81]. The optical response of Ti_3_C_2_T*_x_* can be precisely tuned via surface terminations, which influence interband and intraband electronic transitions. Hydroxylated and fluorinated terminations, which typically accept one electron each, yield similar absorption features in the visible spectrum, whereas oxygen terminations, requiring two electrons, impart distinctive optical signatures [81]. Mechanically, MXenes exhibit excellent flexibility and tensile strength, making them viable candidates for structural composites and robust coatings [76]. Their mechanical behavior is influenced by bonding configurations between metal and nonmetal atoms, such as M–C versus M–N bonds, and the number of layers (n) in the general formula M_n+1_X_n_. For instance, Ti_2_C, characterized by longer Ti–C bonds than Ti_3_C_2_, exhibits different elastic moduli and deformation responses [78]. Surface terminations also significantly affect mechanical strength, with –O-terminated MXenes generally exhibiting superior mechanical robustness relative to their –F- and –OH-terminated counterparts [3,82].

Computational modeling has been pivotal in elucidating the rich structural diversity of MXenes and guiding the discovery of novel compositions [62,83]. To date, six distinct structural categories have been identified: (i) mono-M elements such as Ti_2_CeNb_4_C_3_; (ii) solid solution phases like (Cr, V)_3_C_2_, and (Ti, V)_3_C_2_; (iii) out-of-plane ordered double-M elements such as Mo_2_Ti_2_C_3_ and Mo_2_TiC_2_; (iv) in-plane ordered structures such as (Mo_2/3_Y_1/3_)_2_AlC; (v) vacancy-ordered MXenes (e.g., Mo_1_._33_CT*_x_*); and (vi) randomly disordered vacancy structures (e.g., Nb_1.33_CT*_x_*) [77].

**Figure 3:**
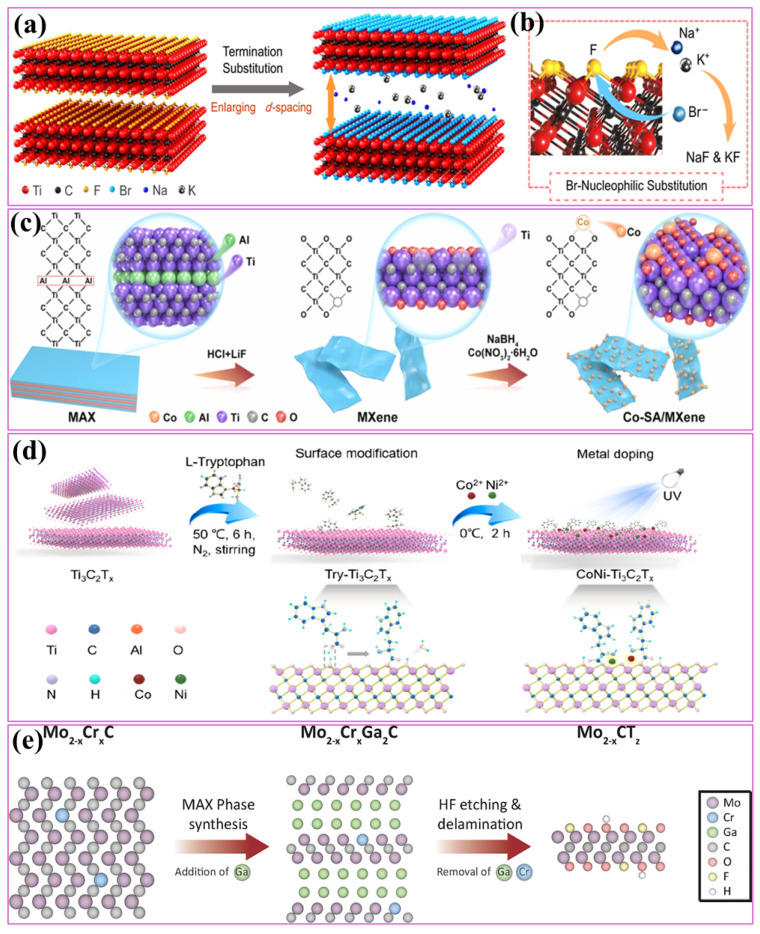
(**a**) Schematic of the preparation of Lewis-basic halide-modified Ti_3_C_2_T*_x_*; (**b**) process of the nucleophilic substitution between desolvated Br^−^ and F atoms (reproduced with permission from [84]); (**c**) schematic of the synthesis of Co-SA/MXene (SA: single atom) (reproduced with permission from [86]); (**d**) schematic illustration of anchoring L-tryptophan at the surface of Ti_3_C_2_T_x_, followed by fabrication of dual-atom CoNiTi_3_C_2_T_x_ composites (reproduced with permission from [87]); (**e**) defect-engineered Mo_2−x_CT_z_ MXene prepared by selective etching and exfoliation of Mo_2−x_Cr_x_Ga_2_C MAX phases, introducing controlled vacancies and strain to modulate electronic structure and catalytic performance (reproduced with permission from [89]).

Surface engineering has proven especially impactful among the strategies to enhance catalytic performance. Techniques such as heteroatom doping, termination, defect engineering, and heterostructure designing have been extensively explored [3,10,34,37]. Surface properties critically influence electrocatalytic activity by modulating the nature and accessibility of active sites [55]. Depending on the chemical environment, MXene surfaces spontaneously acquire termination groups (–O, –OH, –F) during etching [8]. These groups govern hydrophilicity, stability, electronic structure, and catalytic behavior, offering a straightforward route for tailoring material properties. Zhang et al. [84] reported a general method to simultaneously engineer the surface termination and enlarge the interlayer spacing of MXenes by Lewis-basic halides. For this aim, Lewis-basic AlBr_3_/NaBr/KBr and AlI_3_/NaI/KI eutectic molten salts were used for MXene treatment. As shown in Figure 3a, the interlayer spacing of MXene could be enlarged due to the synergism of termination substitution and desolvated cations intercalation (Na^+^ and K^+^) in Lewis-basic halides, and the –F termination could be substituted through reaction with desolvated Br^−^ and I^−^ (Figure 3b). Interestingly, this material showed a capacity almost two-fold higher than pristine Ti_3_C_2_T*_x_* for Li^+^ storage.

Heteroatom doping involves the incorporation of foreign atoms, often transition metals or light elements, into the MXene lattice, thereby altering the electronic charge distribution, band structure, and density of catalytically active sites. Such doping can optimize intermediate adsorption and improve charge transfer kinetics in electrochemical reactions [85]. For instance, anchoring isolated single atoms within the MXene framework prevents aggregation and maximizes exposure of active centers [8]. Xin et al. [86] synthesized Ti_3_C_2_T*_x_* nanosheets by etching Ti_3_AlC_2_ in a mixed solution of HCl and LiF, as shown in Figure 3c. Subsequently, Co-SA/MXene was synthesized using a one-step method involving NaBH_4_ reduction. It exhibited superior efficiency compared to pure MXene. Compared to single-atom catalysts, dual-atom catalysts can provide more flexible configurations for different reaction intermediates, and synergistic effects between two different catalytic sites may accelerate the multi-intermediate reaction kinetics [87]. Zhao et al. [87] introduced a surface-modification strategy of MXene substrates by preabsorbing L-tryptophan molecules, which enabled attachment of dual-atom Co/Ni electrocatalyst at the surface of Ti_3_C_2_T*_x_* by forming N−Co/Ni-O bonds (Figure 3d). The electron delocalization resulting from terminated O atoms on MXene substrates, N atoms in L-tryptophan anchoring moieties, and catalytic metal atoms Co and Ni provides an optimal adsorption strength of intermediates and boosts the HER and OER kinetics. Non-metal dopants, particularly nitrogen introduced via lattice substitution, modulate electronic states effectively and improve catalytic performance [88]. Defect engineering in the form of the intentional creation of defects has been shown to enhance the properties of two-dimensional materials in various applications. Defect engineering, intentionally introducing vacancies and edge sites, constitutes another powerful strategy for catalytic enhancement. Vacancies typically form during etching, even under mild conditions, while edge defects can be generated through dimensional reduction or porosity enhancement processes. Both defects enrich the density of active sites and beneficially modify the electronic characteristics of MXenes, contributing to improved catalytic kinetics [8]. Ronchi et al. explored a simple and reproducible method for introducing random vacancies and pores in Mo-based MXenes. The process is based on alloying Mo_2_Ga_2_C with Cr, then etching Ga and Cr in hydrofluoric acid, resulting in vacancies and vacancy clusters in the MXene sheets (Figure 3e). The Mo_1.87_CT*_z_* derived from Mo_1.87_Cr_0.13_Ga_2_C (6.5 atom % Cr) exhibited excellent electrochemical behavior and suggested that defect concentration can be used to tune the rate capability of the material [89].

**Figure 4:**
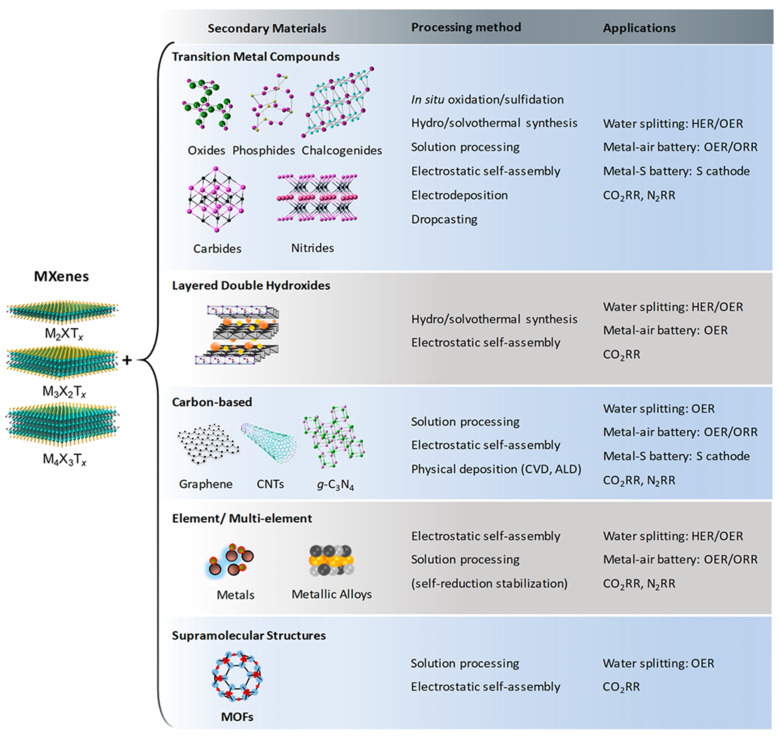
Illustration of three different types of MXene (M_2_XT*_x_*, M_3_X_2_T*_x_*, and M_4_X_3_T*_x_*) combined with secondary materials to form MXene-based composites. Cyan, dark gray, and yellow spheres represent M, X, and T*_x_*. Common synthesis approaches and corresponding catalytic applications are also presented. Reproduced with permission from [101].

Combining MXenes with other nanomaterials and constructing heterostructures constitutes a powerful strategy to further enhance their electrocatalytic performance (Figure 4). Such heterojunctions facilitate efficient charge separation, induce built-in electric fields, and optimize electron–hole transport dynamics. These synergistic effects reduce overpotentials and enhance the catalytic activity and stability of the composite materials [2]. MXenes exhibit excellent compatibility with a wide range of functional materials, including carbon-based nanostructures [90,91], LDHs [92,93], transition metal dichalcogenides (TMDs) [94], metal oxides [95], and MOFs [96,97]. Their rich surface chemistry, combined with their ultralow work function and tunable band alignments, enables seamless interfacial integration and strong electronic coupling with these counterparts, leading to enhanced charge transfer efficiency and stability [85]. In most reported MXene-based heterostructures, the developed composites exhibit outstanding improvements in catalytic activity. This enhancement is attributed to the synergistic interplay of factors, such as accelerated charge transfer kinetics, increased electrochemical surface area, strong interfacial coupling, higher electrochemical stability, ample electrochemically active sites, and high hydrogen binding, along with high dissociation rates [98,99,100]. These advantages position MXene-based heterostructures as promising candidates for the next generation of high-performance electrocatalysts in water splitting and related energy-conversion applications.

## 4. Electrocatalytic Activity of MXene-Based Materials

### 4.1. MXene-Based Electrocatalysts for the HER

High gravimetric energy density and zero-carbon emissions position hydrogen as a key player in future clean energy systems. Among various hydrogen production routes, the HER via electrochemical water splitting stands out for its sustainability and scalability [8]. However, practical implementation critically depends on developing highly active, durable, and cost-effective electrocatalysts to overcome the inherently sluggish HER kinetics. Although Pt remains the benchmark catalyst owing to its near-optimal hydrogen adsorption energy and superior catalytic performance [102], its high cost and scarcity limit widespread application. This challenge has motivated extensive investigation into alternative electrocatalysts, including transition metal compounds, such as oxides, sulfides, phosphides, carbides, and nitrides, and atomically dispersed noble metals.

#### 4.1.1. Pristine MXene

MXenes have garnered significant attention as promising HER electrocatalysts due to their intrinsic hydrophilicity, excellent electrical conductivity, and highly tunable surface chemistry, which facilitates rapid charge transfer and improves the accessibility of catalytically active sites. However, the pristine forms of MXenes generally exhibit modest HER activity, necessitating structural and compositional modifications to enhance their performance. For example, delaminated Mo_2_CT*_x_* MXene operating in acidic electrolyte (0.5 M H_2_SO_4_) exhibits an overpotential of 283 mV at 10 mA cm^−2^ [103], underscoring the need for further optimization. Efforts to improve performance have focused on multielement substitution and alloying within the MXene lattice. Luxa et al. [104] synthesized Mo_2_TiC_2_T*_x_* MXene, demonstrating HER activity with an overpotential of 350 mV at 10 mA cm^−2^, highlighting the role of multielement substitution. Similarly, Snyder et al. [105] synthesized (V_1−y_Mo_y_)_2_CT*_x_* MXenes and observed a significant improvement in HER performance compared to pristine V_2_CT*_x_* (568 mV), with the best-performing composition, (V_0.5_Mo_0.5_)_2_CT*_x_*, achieving an overpotential of 394 mV at 10 mA cm^−2^. This finding highlights the influence of incorporating two different metal centers (V and Mo), which creates synergistic effects that can optimize the electronic structure and surface chemistry, enhance active site availability, and improve charge transfer.

#### 4.1.2. Metals Incorporated into MXene

**Figure 5:**
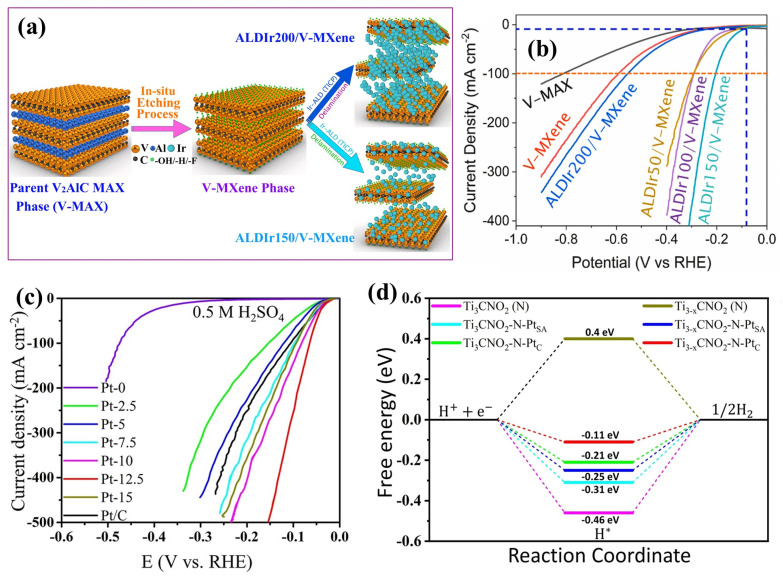
(**a**) Schematic of delaminated V_2_CT*_x_* MXene synthesized by in situ mild etching of V_2_AlC, enabling the development of ALD Ir-functionalized V-MXene heterostructure, and (**b**) LSV curves for HER comparing Ir-ALD cycles (50–200), V_2_AlC, and pristine V_2_CT*_x_* MXene. Reproduced with permission from [4]. (**c**) LSV curves of the MXene, Pt/C, and all Pt-MXene samples with (**d**) calculated free energies for hydrogen evolution on Ti_3_CNO_2_(N) MXene, Ti_3_CNO_2_-N-PtSA, Ti_3_CNO_2_-N-PtC, Ti_3-x_CNO_2_ (N) MXene, Ti_3-x_CNO_2_-N-PtSA, and Ti_3-x_CNO_2_-N-PtC. Reproduced with permission from [106].

Incorporating metal species into MXene frameworks has emerged as a powerful strategy to overcome the intrinsic limitations of pristine MXenes, such as limited active site density, moderate conductivity under certain conditions, and suboptimal ΔG_H*_. Metal incorporation, particularly with noble or transition metals, introduces highly active catalytic centers and modulates the surface electronic structure of MXenes to enhance their overall electrocatalytic performance. For example, Mohapatra et al. [4] utilized atomic layer deposition (ALD) to anchor Ir single atoms/nanoclusters onto V_2_CT*_x_* MXene (Figure 5a), lowering the overpotential to 91 mV at 10 mA cm^−2^, in comparison with V_2_CT*_x_* MXene (271 mV) (Figure 5b). A 2000-cycle CV test at 100 mV s^−1^ showed only a 65 mV increase in overpotential at 10 mA cm^−2^, indicating excellent catalytic stability. Structural durability was further confirmed by a 100 h CA test with negligible current loss, and post-electrolysis characterization revealed no morphological or chemical degradation of the ALD-Ir/V-MXene, attributed to strong Ir–MXene interfacial interactions. In another study, Lei et al. [106] used a self-reduction route to anchor Pt single atoms and clusters onto Ti_3_CNT*_x_* MXene, reducing the overpotential from 329 to 28 mV at 10 mA cm^−2^ in 0.5 M H_2_SO_4_ (Figure 5c). This significant enhancement underscores the pivotal role of Pt in modulating catalytic activity and optimizing hydrogen adsorption–desorption dynamics. Charge density difference calculations show that 1.56 electrons were transferred from the Pt cluster to the MXene in the Ti_3-*x*_CNO_2_–N–Pt_C_, compared to only 0.1, 0.3, and 1.2 electrons in Ti_3_CNO_2_–N–Pt_SA_, Ti_3_CNO_2_–N–Pt_C_, and Ti_3-x_CNO_2_–N–Pt_SA_, respectively. This stronger charge transfer weakened the Pt–H bond strength, improving hydrogen adsorption/desorption and lowering the energy barrier for H_2_ evolution, as confirmed by the improved catalytic kinetics illustrated in Figure 5d. In addition, the catalyst exhibited no potential fluctuation over 23 h at a constant current density of 10 mA cm^−2^.

**Figure 6:**
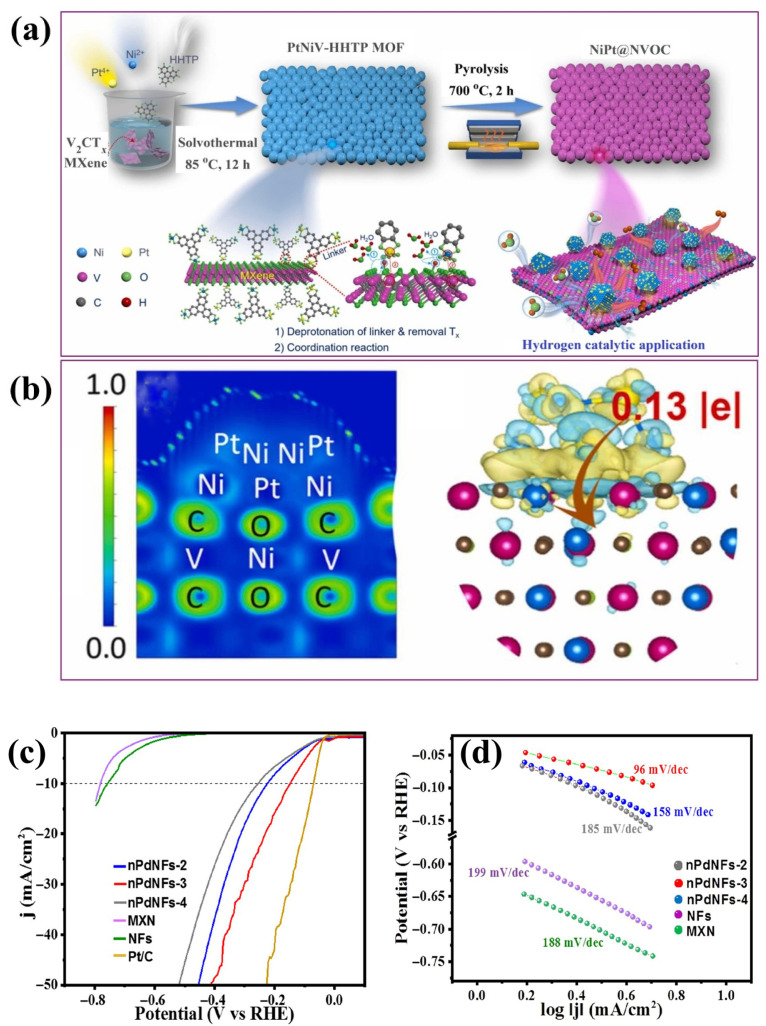
(**a**) Schematic illustrating NiPt@NVOC fabrication via targeted structural transformation and reorganization strategies and (**b**) electron localization function with Bader charge distribution of NiPt@NVOC. Reproduced with permission from [107]. (**c**) LSV curves and (**d**) Tafel plot of the Pd/Ti_3_C_2_ MXene composite, Pt/C, and pristine MXene. Reproduced with permission from [109].

Recent advances included the integration of NiPt onto V_2_CT*_x_* MXene-derived oxycarbide (NiPt@NVOC), as shown in Figure 6a, yielding an overpotential of 11.9 mV and a Tafel slope of 25.8 mV dec^−1^ for the HER reaction [107]. This enhancement is attributed to the synergistic interactions between Ni, Pt, and the MXene (Figure 6b). The catalyst exhibited excellent stability, retaining 98% and 97% of its current response at 10 and 50 mA cm^−2^ over 50 h with negligible degradation. SEM analysis confirmed a well-preserved 2D micro-spherical morphology post-HER, and the XRD pattern retained its original crystalline structure without the emergence of secondary phases. Charge density difference analysis revealed a charge transfer of 0.13 electrons from NiPt clusters to the NVOC surface, promoting strong interfacial bonding and uniform cluster dispersion. This enhanced electron redistribution correlates with a significant reduction in the Tafel slope compared to individual components, NiPt (58.8 mV dec^−1^), Pt@VOC (74.9 mV dec^−1^), and Ni@NVOC (170.2 mV dec^−1^). The strengthened electronic coupling at the NiPt@NVOC interface facilitated faster reaction kinetics by optimizing hydrogen adsorption energy and accelerating charge carrier transport during HER. Moreover, the projected density of state (PDOS) showed a d-band center of −2.28 eV, higher than NiPt alone (−2.46 eV), indicating stronger metal–adsorbate interactions and enhanced electron mobility. The Pt d-states in NiPt@NVOC are positioned near the optimal value of −1.929 eV, which correlates with favorable ΔG_H*_ and reduced desorption energy, thus facilitating efficient H_2_ evolution. Similarly, uniform Ni nanoparticle deposition on Mo_2_TiC_2_T*_x_* MXene via vacuum treatment achieved an overpotential of 242.5 mV, significantly outperforming pristine Mo_2_TiC_2_T*_x_* (358.6 mV) at 10 mA cm^−2^ [108]. Moreover, the LSV curves after 75 h closely matched the initial scan, confirming the catalytic stability. Raj et al. [109] further demonstrated the effectiveness of noble metal modification by synthesizing palladium (Pd)-decorated Ti_3_C_2_ nanoflowers via a hydrothermal approach conducted at 120 °C for 10 h. The Pd/Ti_3_C_2_ composite exhibited an overpotential of 149 mV at 10 mA cm^−2^ and a Tafel slope of 96 mV dec^−1^ (Figure 6c,d) compared to the poor efficiency of unmodified Ti_3_C_2_ (761 mV), indicating a substantial catalytic enhancement through Pd incorporation. The enhanced performance was attributed not only to the catalytic role of Pd but also to the nanoflower morphology generated by the hydrothermal treatment, which promoted higher surface area and intimate Pd–MXene interfacial contact. These structural features facilitated efficient charge transfer and hydrogen adsorption/desorption dynamics. Stability assessment showed that the catalyst initially exhibited a slight decrease in potential over the first 2 h, followed by plateau behavior up to 24 h (retained 94% of its potential) with a minor initial drift (~70 μV s^−1^). These findings highlighted the promise of MXene-based materials for HER processes, particularly through surface and compositional tuning. Metal incorporation significantly enhances performance, positioning MXenes as viable candidates for scalable hydrogen production.

#### 4.1.3. Metal-Incorporated MXene/Carbon Hybrids

**Figure 7:**
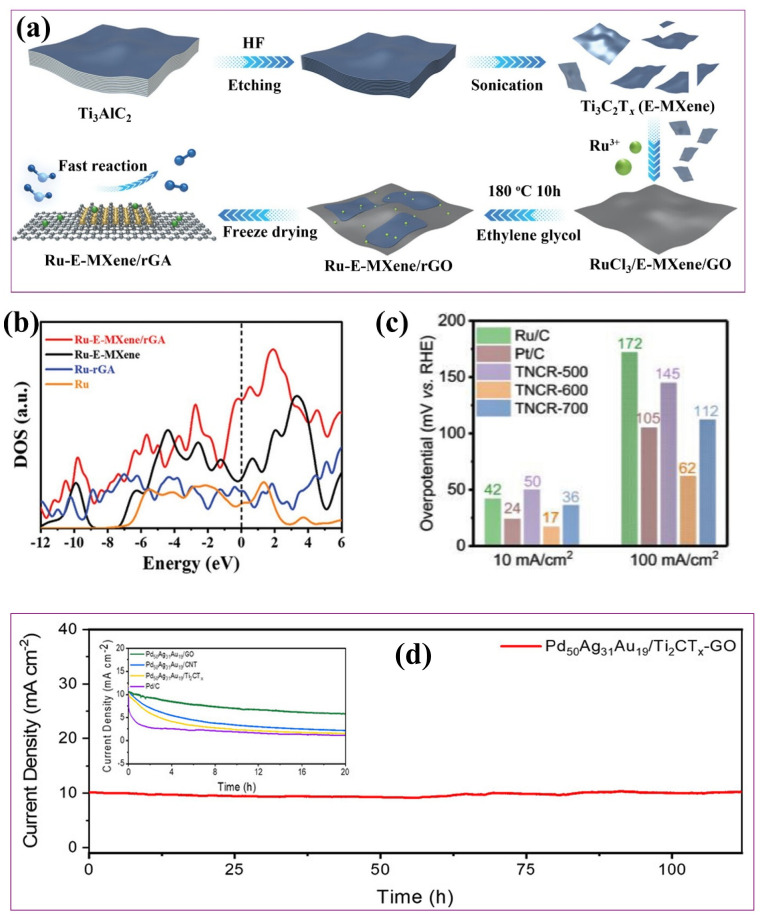
(**a**) Schematic of Ru-E-MXene/rGA synthesis with (**b**) TDOS comparison for Ru-E-MXene/rGA, Ru-E-MXene, Ru-rGA, and Ru, illustrating electronic structure modifications. Reproduced with permission from [111]. (**c**) The overpotentials of Ti_3_C_2_Tx-N/C-Ru (TNCR) at different calcination temperatures, Ru/C, and Pt/C at different current densities. Reproduced with permission from [112]. (**d**) CA of PdAgAu alloy embedded in Ti_2_CT*_x_* MXene/graphene oxide electrocatalysts at 10 mA cm^−2^. Reproduced with permission from [113].

Integrating MXenes with carbon-based materials and functionalizing the resulting composites with active metal species has emerged as a promising strategy to further enhance their electrocatalytic HER performance. Carbonaceous materials like graphene effectively prevent MXene restacking, improve conductivity, and impart structural robustness. Xue et al. [110] demonstrated a scalable approach for synthesizing 0D/2D heterojunctions by anchoring graphene quantum dots (GQDs) onto Ti_3_C_2_T*_x_* MXene. Compared to pristine Ti_3_C_2_T*_x_* MXene, which exhibited an overpotential of 551 mV at 10 mA cm^−2^ and a Tafel slope of 179 mV dec^−1^, the GQDs/Ti_3_C_2_T*_x_* composite significantly improved HER activity, lowering the overpotential to 260 mV and the Tafel slope to 89 mV dec^−1^. A synergistic tri-component interface is established when metal species are incorporated into these MXene-carbon frameworks. The carbon scaffold ensures the uniform dispersion and stabilization of metal atoms or clusters, while the metals contribute high catalytic activity. Additionally, the MXene layers facilitate rapid electron transport and provide mechanical support. This integrated architecture modulates the local electronic environment and optimizes the energetics of hydrogen adsorption, collectively advancing HER kinetics. Numerous studies have demonstrated the effectiveness of integrating metal atoms or clusters into MXene-carbon hybrid frameworks to enhance HER activity. Yu et al. [111] synthesized a Ru nanocluster-decorated Ti_3_C_2_T*_x_* MXene/reduced graphene oxide composite via a glycol reduction method (Figure 7a), achieving an overpotential of 42 mV at 10 mA cm^−2^ and a Tafel slope of 36.6 mV dec^−1^ in 1.0 M KOH electrolyte, which was superior to both Ru-Ti_3_C_2_T*_x_* MXene (66 mV) and Ru-reduced graphene oxide (70 mV). The Ru-MXene/rGA catalyst also demonstrated excellent catalytic stability, with only a 4 mV increase in overpotential after 1000 CV cycles and a negligible shift in CV curves. This enhancement was ascribed to strong interfacial coupling, which modulated the local electronic structure, facilitated charge transfer, and optimized hydrogen adsorption–desorption kinetics, as evidenced by the increased total density of states (TDOS) near the Fermi level (Figure 7b).

Similarly, Wu et al. [112] developed a ternary hybrid of Ru nanoparticles anchored on N-doped carbon/Ti_3_C_2_T_x_ MXene (TNCR), achieving an ultralow overpotential of 17 mV at 10 mA cm^−2^ at a calcination temperature of 600 °C (Figure 7c). Impressively, the catalyst maintained high electrochemical stability even after 20,000 CV cycles and retained a stable potential over 160 h of continuous HER operation at 400 mA cm^−2^. The composite architecture promoted uniform Ru dispersion, enhanced electron transport, and favorable hydrogen adsorption energetics. In another study, Sun et al. [113] reported a PdAgAu alloy embedded in Ti_2_CT_x_ MXene/graphene oxide, which exhibited a low overpotential of 90 mV at 10 mA cm^−2^ and demonstrated robust stability over 112 h, retaining 97% of the initial current density (Figure 7d). This exceptional performance was attributed to electronic modulation and interfacial synergy. These findings underscore the potential of MXene-carbon–metal hybrids as high-performance and scalable HER electrocatalysts.

#### 4.1.4. Metal Oxide–MXene Composites

**Figure 8:**
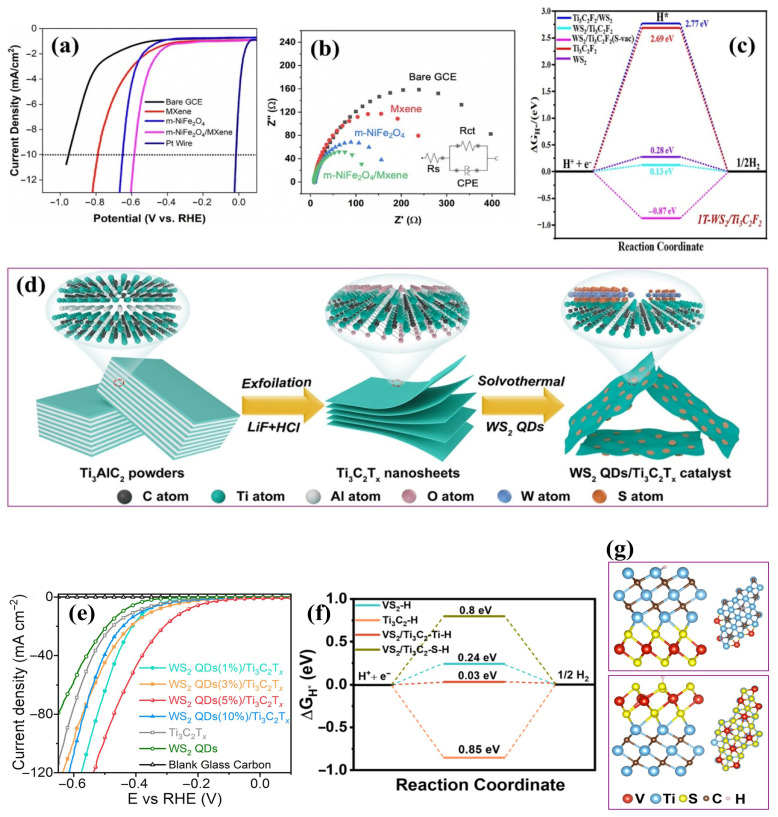
(**a**) LSV curves and (**b**) Nyquist plots of glassy carbon, m-NiFe_2_O_4_, m-NiFe_2_O_4_/MXene, MXene, and Pt wire using 1 M NaOH. Reproduced with permission from [115]. (**c**) ΔG_H*_ profile at the active site on the WS_2_, Ti_3_C_2_T_2_, WS_2_−Ti_3_C_2_T_2_, Ti_3_C_2_T_2_/WS_2_, and WS_2_−Ti_3_C_2_T_2_(S-vac) surfaces. Reproduced with permission from [100]. (**d**) Schematic of preparation of the WS_2_ QDs/Ti_3_C_2_T_x_ MXene heterostructure and (**e**) LSV curves of WS_2_ QDs/Ti_3_C_2_T_x_ with different WS_2_ QD contents, Ti_3_C_2_T_x_, and WS_2_ QDs. Reproduced with permission from [116]. (**f**) Calculated H* adsorption-free energy for VS_2_, Ti_3_C_2_, and VS_2_/Ti_3_C_2_ with a (**g**) VASP model of hydrogen adsorption by VS_2_/Ti_3_C_2_−Ti-H and VS_2_/Ti_3_C_2_−S-H. Reproduced with permission from [117].

Integrating metal oxides with MXenes has become a practical approach to boost HER electrocatalysis. Oxides such as RuO_2_, Co_3_O_4_, NiO, and Fe_2_O_3_ exhibit favorable redox properties, chemical robustness, and tunable electronic structures, collectively contributing to their enhanced catalytic activity. When interfaced with MXenes, they provide additional active sites, modulate charge distributions, and accelerate reaction kinetics. Concurrently, MXenes act as highly conductive 2D scaffolds that suppress oxide agglomeration, promote electron transfer, and increase surface accessibility. These composite architectures overcome the limitations of individual components through interface engineering and electronic coupling. Yin et al. [114] synthesized an amorphous CoSnO_3_/Ti_3_C_2_T*_x_* MXene composite by calcining Ti_3_C_2_T*_x_* MXene@CoSn(OH)_6_ under Ar at 350 °C. It revealed an overpotential of 45 mV at 10 mA cm^−2^ and a Tafel slope of 51 mV dec^−1^ for the HER process in 1.0 M KOH electrolyte. The enhanced catalytic performance was attributed to interfacial charge redistribution, as revealed by an analysis of charge density differences. Electrons were transferred from Co to Sn atoms. Sn sites with a two-oxygen coordination, positioned along the charge transfer pathway, retained charge neutrality and exhibited balanced *H adsorption energy. In contrast, Sn sites that accumulated excess electrons displayed overly strong *H adsorption, while Co centers became electron-deficient. The electronically neutral Sn sites facilitated moderate hydrogen binding, enhancing HER kinetics. This electronic optimization correlated with the lower observed Tafel slope of 51 mV dec^−1^ for the CoSnO_3_/Ti_3_C_2_T*_x_* MXene composite, compared to 89 mV dec^−1^ for CoSnO_3_ and 111 mV dec^−1^ for MXene. Similarly, Ghaemmaghami et al. [93] anchored Co_3_O_4_ microspheres onto Ti_3_C_2_T*_x_* sheets via thermal decomposition of cobalt glycerate precursors at 350 °C for 2 h. This method promoted the formation of well-defined Co_3_O_4_ structures while ensuring strong interfacial bonding with the MXene. The controlled decomposition facilitated uniform dispersion of Co_3_O_4_ particles across the surface, effectively preventing MXene restacking and exposing more active sites. The resulting hybrid material exhibited an overpotential of 118 mV at 10 mA cm^−2^ and demonstrated excellent stability over 24 h in alkaline media, with polarization curves changing by just 19 mV. The Co_3_O_4_ particles were uniformly dispersed across the MXene surface, significantly preventing MXene restacking and offering abundant catalytic sites. In another study, Khalil et al. [115] prepared a mesoporous NiFe_2_O_4_/Ti_3_C_2_T*_x_* MXene composite using a nanocasting method with SBA-15 silica as a hard template. This approach enabled precise control over pore structure and morphology, ensuring uniform distribution and intimate contact at the NiFe_2_O_4_–MXene interface, which facilitated more efficient charge transfer and electrolyte accessibility. The hybrid material exhibited an overpotential of 584 mV in basic conditions (1.0 M NaOH), significantly lower than that of pristine Ti_3_C_2_T*_x_* MXene (650 mV) (Figure 8a). This improvement was primarily attributed to the synergistic integration of the high surface area of mesoporous NiFe_2_O_4_ with the excellent conductivity of the MXene, as confirmed by Nyquist plots in Figure 8b.

#### 4.1.5. Metal Sulfide–MXene Composites

Incorporating metal sulfides with MXenes has proven effective for enhancing HER activity. Metal sulfides offer distinct advantages, including abundant redox-active sites, tunable electronic configurations, and intrinsically favorable hydrogen adsorption-free energies, positioning them as efficient HER catalysts. When coupled with MXenes, the resulting heterostructures exhibit expanded electrochemical surface area and enhanced structural integrity. The strong interfacial interaction between components suppresses sulfide aggregation and facilitates rapid charge transfer, accelerating HER kinetics. Rasool et al. [100] synthesized a 2D/2D WS_2_-Ti_3_C_2_T*_x_* MXene composite by uniformly growing WS_2_ nanopetals onto MXene nanosheets via a one-step solvothermal route conducted at 200 °C for 24 h. This process promoted homogeneous nucleation and vertical alignment of WS_2_ on the MXene surface, ensuring intense interfacial contact and preventing restacking of the MXene layers. The heterostructure delivered an overpotential of 66 mV at 10 mA cm^−2^, a Tafel slope of 46.7 mV dec^−1^ in 1.0 M KOH, and excellent durability over 50 h at 100 mA cm^−2^, with only a slight variation in overpotential (9.53%). Moreover, post-HER characterization confirmed the crystalline phase and morphology retention, validating the structural integrity of the composite. The enhancement was attributed to strong interfacial coupling between WS_2_ and Ti_3_C_2_T*_x_*, which increased active site density and accelerated charge transfer kinetics. Density functional theory (DFT) calculations confirmed that the composite material exhibited a nearly optimal ΔG_H*_ of 0.13 eV (Figure 8c). Charge density difference calculations further revealed pronounced electron accumulation near S atoms in the WS_2_–Ti_3_C_2_F_2_ hybrid, indicating electron transfer from WS_2_ to Ti_3_C_2_F_2_. This interfacial charge redistribution modified the local electronic structure and weakened the H adsorption energy, leading to more favorable *H intermediate formation and desorption. As a result, the improved electronic landscape facilitated faster proton–electron transfer kinetics during HER, which quantitatively correlates with the reduced Tafel slope of the composite. In another study, Xie et al. [116] fabricated WS_2_ QDs anchored onto Ti_3_C_2_T*_x_* via a solvothermal assembly method conducted at 120 °C for 12 h (Figure 8d). This mild solvothermal process facilitated uniform nucleation and growth of WS_2_ QDs on the MXene surface, promoting abundant heterointerface formation and preventing QD aggregation. The resulting composite material exhibited an overpotential of 247 mV at 10 mA cm^−2^ in acidic electrolytes (0.5 M H_2_SO_4_) (Figure 8e) and retained high stability over a 24 h CP test. Furthermore, the as-recorded LSV curve remained nearly unchanged after 2000 CV cycles. The uniform dispersion of WS_2_ QDs enabled the formation of abundant heterointerfaces, which enhanced electronic conductivity and led to synergistic effects during electrocatalysis. In another study, Zhao et al. [117] designed a VS_2_/Ti_3_C_2_ MXene heterostructure via a hydrothermal procedure, achieving an exceptional overpotential of 33 mV and a Tafel slope of 25.9 mV dec^−1^ in an acidic electrolyte (0.5 M H_2_SO_4_). The formation of interfacial Ti–S bonds was instrumental in modulating the local electronic environment (Figure 8f,g), optimizing hydrogen adsorption, and enhancing interfacial conductivity. In addition, ΔG_H*_ was calculated to further rationalize the observed HER performance. The VS_2_/Ti_3_C_2_–Ti site exhibited a nearly thermoneutral ΔG_H*_ value of 0.03 eV, markedly lower than that of pristine VS_2_ (0.24 eV) and Ti_3_C_2_ (−0.85 eV), which explains its superior catalytic activity.

**Figure 9:**
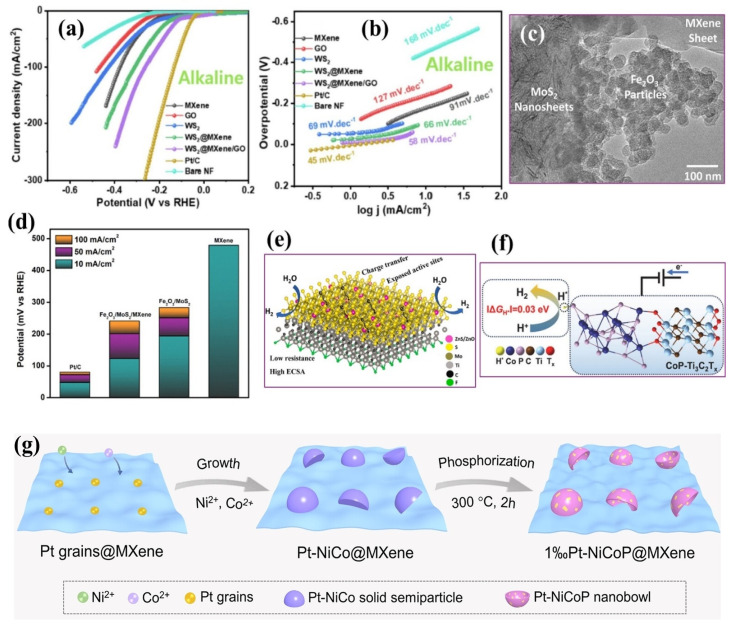
(**a**) LSV curves and (**b**) Tafel plots comparing nickel foam (NF), Pt/C, MXene, GO, WS_2_, WS_2_@MXene, and WS_2_@MXene/GO. Reproduced with permission from [118]. (**c**) TEM image of Fe_2_O_3_/MoS_2_/Ti_3_C_2_T*_x_* MXene ternary composite with a (**d**) comparison of the overpotential at different current densities. Reproduced with permission from [119]. (**e**) Proposed HER mechanism on the ZnS-ZnO-MoS_2_-MXene electrocatalyst. Reproduced with permission from [120]. (**f**) Schematic of the HER process on a CoP-Ti_3_C_2_T*_x_* hybrid interface. Reproduced with permission from [121]. (**g**) Schematic illustration of the growth of 1%Pt on NiCoP@Ti_3_C_2_T*_x_* MXene. Reproduced with permission from [122].

Furthermore, Hussain et al. [118] developed a porous WS_2_-embedded Ti_3_C_2_T*_x_* MXene/graphene oxide hybrid material through a hydrothermal self-assembly approach. This ternary architecture exhibited rapid charge transfer, a high density of exposed active sites, and robust structural integrity. It achieved a remarkable overpotential of 45 mV at 10 mA cm^−2^ and a Tafel slope of 58 mV dec^−1^ in alkaline electrolytes (Figure 9a,b), with ΔG_H*_ of −0.46 eV, surpassing its components, WS_2_ (98 mV, 0.66 eV), Ti_3_C_2_T*_x_* MXene (191 mV, −1.11 eV), and GO (242 mV, 1.13 eV). Additionally, the nearly overlapping LSV curves before and after 24 h and the unchanged FE-SEM morphology confirmed the catalyst’s structural robustness under prolonged operation. These results underscore the significance of interfacial engineering and multicomponent synergy in enhancing MXene-sulfide-based HER electrocatalysts.

The co-integration of metal oxides and sulfides onto MXene scaffolds has emerged as an effective strategy to enhance HER electrocatalysis. Metal oxides contribute to chemical robustness and diverse redox behavior, whereas metal sulfides offer intrinsically high catalytic activity and favorable hydrogen adsorption energetics. When simultaneously interfaced within an MXene matrix, these two components establish interfacial heterojunctions that enable synergistic electronic interactions and charge redistribution. This coupling accelerates HER kinetics and enhances the stability and activity of the catalytic interface. Meanwhile, the MXene support is a highly conductive 2D framework that mitigates the aggregation of active phases, promotes rapid electron transport, and maintains structural cohesion. Such ternary oxide–sulfide–MXene hybrids often outperform their individual or binary analogs due to the combined advantages of interfacial engineering and compositional synergy. Sumanth et al. [119] synthesized a Fe_2_O_3_-MoS_2_/Ti_3_C_2_T*_x_* composite material using a hydrothermal method, resulting in the uniform deposition of both Fe_2_O_3_ and MoS_2_ on the MXene surface (Figure 9c). The resulting catalyst exhibited a significantly reduced overpotential of 194.1 mV at 10 mA cm^−2^ and a Tafel slope of 102 mV dec^−1^ in acidic conditions (0.5 M H_2_SO_4_) (Figure 9d), markedly surpassing the performance of Fe_2_O_3_ (478 mV) and MoS_2_ (256 mV). Post-stability evaluation revealed slight overpotential increases after 1000 CV cycles and minimal current decay over a 14 h CA test. XRD and surface analysis indicated minor peak broadening and shifts, likely due to strain from acidic cycling, but overall phase integrity was maintained. This enhancement was attributed to the electronic synergy between oxide and sulfide phases, which modulated the local charge distribution and increased the density of catalytically active sites. Meanwhile, the MXene provided a conductive network for efficient charge transfer. In another example, Rasool et al. [120] fabricated a ZnS-ZnO/MoS_2_/Ti_3_C_2_T*_x_* hybrid using a one-pot hydrothermal synthesis. This multi-phase system exhibited an overpotential of 327.6 mV at 10 mA cm^−2^ in acidic electrolyte (0.5 M H_2_SO_4_) and stable operation over 50 h. The high performance was attributed to the formation of multi-phase ZnS-ZnO-MoS_2_ heterojunctions anchored on the highly conductive Ti_3_C_2_T*_x_* MXene, as illustrated in Figure 9e. This configuration facilitated abundant catalytic interfaces, enhanced H* adsorption/desorption kinetics, and reinforced the structural stability of the catalyst under operational conditions.

#### 4.1.6. Metal Phosphide–MXene Composites

Coupling metal phosphides with MXenes has recently attracted growing interest as a potent route for constructing advanced HER electrocatalysts with enhanced activity and durability. Metal phosphides, such as Ni_2_P and CoP, are recognized for their intrinsic catalytic activity, conductivity, and thermodynamically favorable hydrogen adsorption energies. However, practical limitations hinder their standalone application, including nanoparticle agglomeration, suboptimal dispersion, and limited long-term operational stability. Integrating these phosphides with MXenes offers a robust platform to address these challenges. The 2D MXene support provides a chemically stable and highly conductive matrix with a large specific surface area, facilitating the uniform anchoring of phosphide nanostructures and thereby enhancing electron mobility, while promoting the efficient exposure and utilization of catalytic sites. The resulting phosphide–MXene heterostructures exhibit strong interfacial interactions, accelerated charge transfer dynamics, and superior catalytic performance across a wide pH range. Sun et al. [121] synthesized a CoP/Ti_3_C_2_T*_x_* composite via a hydrothermal reaction followed by an in situ phosphidation. The catalyst demonstrated a significantly reduced overpotential of 135 mV at 10 mA cm^−2^, a Tafel slope of 48 mV dec^−1^, and maintained its function for 50 h CP without significant attenuation (99%) in 0.5 M H_2_SO_4_. In contrast, the individual Ti_3_C_2_T*_x_* MXene and CoP exhibited significantly higher overpotentials of 449 and 199 mV, respectively, and significantly larger Tafel slopes of 350 and 107 mV dec^−1^. This performance enhancement was attributed to synergistic effects at the CoP–MXene heterointerface (Figure 9f), facilitating charge redistribution and electronic structure modulation. Bader charge analysis confirmed that upon heterojunction formation, the total electron count in the system decreased from 168 electrons in isolated CoP(110) to 166.86 electrons in the CoP/MXene composite, indicating net charge transfer from CoP to MXene. This interfacial electron depletion at the CoP sites shifted its d-band center downward from −1.552 to −1.741 eV relative to the Fermi level, weakening the hydrogen adsorption strength and promoting more efficient hydrogen desorption. These electronic adjustments effectively optimized the H_2_ evolution pathway, lowering the energy barrier for HER and quantitatively explaining the observed reduction in Tafel slope in the CoP–MXene composite. In another study, Niu et al. [122] developed a Pt–NiCoP/Ti_3_C_2_T*_x_* MXene catalyst, wherein a Pt–NiCo alloy precursor was anchored on the MXene surface and converted into nanobowls via a controlled phosphidation process with minimal Pt doping (1.0 mol%) (Figure 9g). The composite material achieved an overpotential of 26.5 mV at 10 mA cm^−2^ and a Tafel slope of 38.6 mV dec^−1^ in a basic electrolyte (1.0 M KOH), substantially outperforming the undoped NiCoP counterpart (75.2 mV). The exceptional catalytic performance was ascribed to forming well-defined Pt–NiCoP/MXene heterointerfaces, which modulated the local electronic structure, enhanced charge transport, and maximized the accessibility of electroactive sites. Moreover, the incorporation of Pt induced a shift toward lower-valence states of Ni and Co, which markedly boosted catalytic activity by reducing the water dissociation energy barrier (E_a_) by approximately 43%, thereby accelerating the Volmer step under alkaline conditions.

Table 3 provides a comparative summary to quantitatively assess the impact of structural and compositional modifications on the HER performance of MXene-based electrocatalysts. It indicates representative ΔG_H*_ values and overpotentials at 10 mA cm^−2^ for various systems, emphasizing the improvements achieved through hybridization, heteroatom doping, and interfacial engineering. This comparative analysis demonstrates that pristine MXenes generally exhibit suboptimal HER activity due to unfavorable ΔG_H*_ values and high overpotentials. In contrast, engineered MXene-based composites, particularly those incorporating noble or transition metal species, exhibit significantly improved catalytic performance, with ΔG_H*_ values approaching thermoneutrality and substantially reduced η_10_. These results underscore the pivotal role of interfacial engineering and the incorporation of guest-active species in fully realizing the electrocatalytic potential of MXenes.

The catalytic performance of MXene-based electrocatalysts is strongly dependent on the electrolyte pH, as HER kinetics and interfacial charge-transfer mechanisms differ markedly across acidic, neutral, and alkaline environments. Achieving stable and efficient HER activity over a wide pH range is thus essential for practical and versatile water-splitting applications. Lei et al. [106] reported that a Ti_3_CNT*_x_* MXene-supported catalyst modified with Pt clusters exhibited overpotentials of 28 mV in 0.5 M H_2_SO_4_ (acidic), 32.8 mV in 1.0 M KOH (alkaline), and 366.4 mV in 0.5 M K_2_SO_4_ (neutral) at 10 mA cm^−2^, alongside consistently favorable Tafel slopes and mass activities under all conditions [106]. These results highlight the robustness of the Pt–MXene interface, which maintains high catalytic activity and structural integrity despite changes in electrolyte pH. Similarly, NiPt nanostructures confined within MXene-derived oxycarbide nanospheres (NiPt@NVOC) demonstrated excellent pH tolerance, requiring overpotentials of only 11.9 mV and 35.6 mV at 10 mA cm^−2^ in acidic and alkaline media, respectively, with corresponding Tafel slopes of 25.8 and 31.2 mV dec^−1^ [107]. This enhanced activity is attributed to partially negatively charged Pt centers facilitating water dissociation and favorable hydrogen adsorption. Although a moderate increase in Tafel slope was observed in alkaline conditions, likely due to inherently slower water dissociation kinetics, the catalyst sustained high performance without significant degradation. Moreover, none of the reported surface modifications, such as cluster dispersion, heterostructure confinement, or MXene support, showed substantial loss of catalytic efficiency across pH variations. These findings highlight that rational surface engineering strategies can effectively endow MXene-based HER catalysts with broad electrochemical resilience and high activity across diverse pH environments. Such robustness is crucial for implementing scalable and versatile water-splitting technologies.

Surface terminations and intrinsic structural defects profoundly influence the electrocatalytic performance of MXene-based HER catalysts. Surface functional groups, such as –O, –OH, and –F terminations, tune the electronic configuration and hydrogen adsorption characteristics of MXenes, thereby modulating the reaction kinetics. Among these, –O terminations generally correlate with more favorable ΔG_H*_ values, enhancing HER activity by facilitating effective hydrogen adsorption and evolution [123]. However, an excessive density of oxygen terminations can result in overly strong hydrogen binding, which hampers the desorption of hydrogen intermediates and ultimately diminishes catalytic efficiency. Conversely, MXenes possessing mixed surface terminations, especially those containing –F groups, commonly exhibit reduced electron density near the Fermi level. This reduction weakens hydrogen adsorption, shifting ΔG_H*_ closer to the thermoneutral optimum (~0 eV), thereby achieving a balanced adsorption–desorption dynamic that optimizes HER kinetics. Despite this advantage, F-terminated MXenes suffer from limited electrochemical stability under HER conditions. The surface fluorine atoms may react with protons, forming HF, leading to progressive fluorine loss and consequent degradation in catalyst durability [104]. In addition to surface chemistry, atomic-scale intrinsic defects, such as metal vacancies and edge sites, play a pivotal role by creating localized electronic states that act as additional catalytic centers. These defects are particularly beneficial in alkaline HER environments, where they can lower activation barriers for hydrogen adsorption and promote the critical initial water dissociation step [106]. DFT calculations leveraging electronic descriptors, such as d-band center shifts and ΔG_H*_, have demonstrated that while surface terminations predominantly regulate the chemical reactivity on basal planes, atomic-scale defects enhance charge localization and accelerate reaction kinetics at edge- and vacancy-rich regions. Furthermore, the interfacial interaction between MXenes and guest species, including reactants, intermediates, and electrolyte ions, is highly sensitive to termination chemistry. This sensitivity influences interfacial charge transfer processes and hydrogen binding strength at the catalyst–electrolyte interface [121], which are crucial for sustaining high catalytic activity and stability. Therefore, rational catalyst design strategies must decouple and systematically optimize surface functionalization and defect engineering effects. Such combined approaches are critical to unlocking the full potential of MXene-based HER electrocatalysts by achieving favorable electronic structures, optimal adsorption energies, enhanced active site density, and robust electrochemical stability.

### 4.2. MXene-Based Electrocatalysts for the OER

The OER is a pivotal half-reaction in electrochemical energy-conversion technologies, including water electrolysis [124], metal–air batteries [125], and reversible fuel cells [126]. However, practical OER implementation is obstructed by intrinsically sluggish four-electron transfer kinetics and high overpotentials required to drive O–O bond formation. Conventional state-of-the-art OER electrocatalysts, such as noble metal oxides (IrO_2_ and RuO_2_) [127], exhibit excellent activity but face challenges related to high cost, scarcity, and insufficient stability under harsh alkaline/acidic environments. MXenes have recently gained attention as promising platforms for OER catalysis [128], owing to their intrinsic electrical conductivity, hydrophilic surfaces, tunable surface terminations, and compositional versatility. While pristine MXenes tend to show limited OER activity because of insufficient active sites, their significant advantage lies in serving as highly conductive, chemically stable supports for anchoring catalytically active species. Integrating transition metal oxides, hydroxides, phosphides, and other active phases onto MXenes enhances OER performance via synergistic effects, improving active site dispersion, accelerating electron transport, and reinforcing structural integrity under oxidative conditions. Furthermore, tailoring MXene surface chemistry and engineering defects can modulate electronic structures as well as optimize key OER intermediate adsorption and desorption energetics (*OH, *O, and *OOH), thus boosting catalytic kinetics [129]. These advancements highlight the potential of MXene-based hybrid architecture in the rational design of high-efficiency OER catalysts for next-generation energy systems.

#### 4.2.1. Metal Oxide–MXene Composites

**Figure 10:**
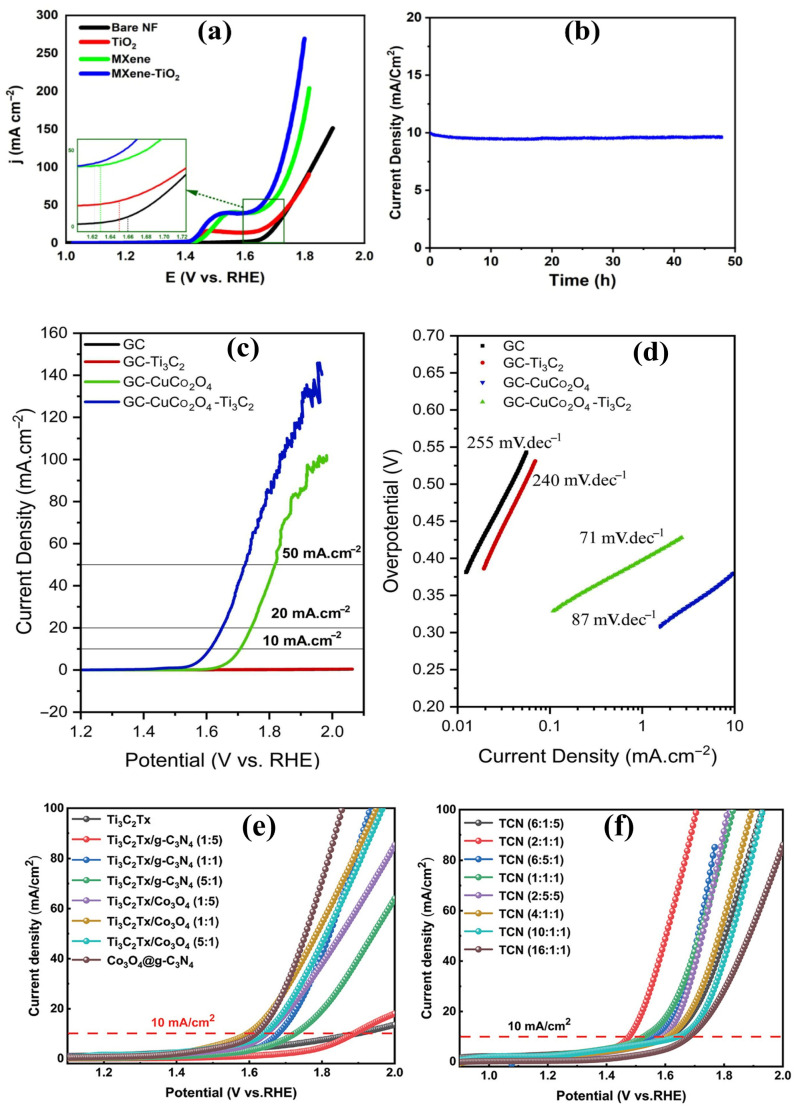
(**a**) LSV curves of NF, MXene, TiO_2_, and MXene-TiO_2_ nanocomposites with a (**b**) CA curve of a V_2_C/TiO_2_ nanocomposite at 10 mA cm^−2^. Reproduced with permission from [131]. (**c**) LSV curves and (**d**) Tafel plots for the GC, Ti_3_C_2_T*_x_*/GC, CuCo_2_O_4_/GC, and CuCo_2_O_4_/Ti_3_C_2_T*_x_*/GC electrodes. Reproduced with permission from [132]. (**e**,**f**) LSV curves of MXene, Co_3_O4/g-C_3_N_4_, and Co_3_O_4_/g-C_3_N_4_/Ti_3_C_2_T*_x_*. Reproduced with permission from [35].

Combining transition metal oxides with MXenes has emerged as a highly effective strategy for enhancing OER activity. Transition metal oxides, such as NiO and Co_3_O_4_, inherently possess favorable redox chemistry and the ability to stabilize oxygenated intermediates but suffer from poor electrical conductivity and limited surface area, hampering charge transfer and access to active sites. In this context, MXenes offer a conductive and hydrophilic matrix to disperse and stabilize oxide nanostructures, overcoming these limitations and enabling improved charge transport and durability. Several recent studies have demonstrated the effectiveness of oxide–MXene composites in accelerating OER kinetics through synergistic interfacial engineering. Raj et al. [130] synthesized Ni_2_O_3_-doped Ti_3_C_2_T*_x_* MXene heterostructures via a hydrothermal approach. The optimized catalyst exhibited an overpotential of 192 mV at 20 mA cm^−2^ and a Tafel slope of 121 mV dec^−1^ in 1.0 M KOH. The LSV stability test indicated that the overpotential increased by only 9 mV after 3000 cycles, confirming the catalyst’s excellent durability and operational stability. The superior catalytic behavior was attributed to nickel oxide-induced band structure modulation, which reduced the bandgap energy and enhanced electronic conductivity. Additionally, Ni_2_O_3_ doping introduced new catalytically active sites with a strong affinity for OER intermediates, facilitating electron transfer and improving reaction kinetics. In another study, Zaka et al. [131] prepared a TiO_2_-decorated V_2_C MXene through a solvothermal method conducted at 120 °C for 12 h. This process enabled uniform nucleation and growth of TiO_2_ nanoparticles across the V_2_C layers, yielding a well-dispersed hybrid structure with intimate interfacial contact. The composite exhibited an overpotential of 425 mV at 50 mA cm^−2^, and maintained robust stability for 48 h CA, with only a slight decline in current density (Figure 10a,b). The improved activity in comparison to TiO_2_ can be mainly ascribed to stabilizing oxygen species at the interface and reducing charge-transfer resistance. Similarly, Ghorbanzadeh et al. [132] fabricated a CuCo_2_O_4_/Ti_3_C_2_T*_x_* MXene heterostructure via a two-step process combining solvothermal synthesis at 150 °C for 3 h followed by post-annealing at 300 °C for 2 h. This stepwise thermal treatment ensured the uniform growth of CuCo_2_O_4_ on the MXene surface and enhanced interfacial contact, promoting stable crystallinity and optimized electron pathways. The composite delivered an overpotential of 380 mV at 10 mA cm^−2^ and a Tafel slope of 71 mV dec^−1^ (Figure 10c,d), markedly outperforming pristine CuCo_2_O_4_ (470 mV). The enhanced activity was attributed to the strong electronic coupling between CuCo_2_O_4_ and Ti_3_C_2_T*_x_* MXene, facilitating rapid charge transfer and the more efficient generation of reactive intermediates during the OER.

Incorporating multifunctional components into ternary hybrid composites further boosts OER activity by combining complementary physicochemical properties to maximize catalytic reactivity, accelerate charge separation, and enhance long-term stability. Li et al. [35] constructed a Co_3_O_4_/g-C_3_N_4_/Ti_3_C_2_T*_x_* heterostructure through a two-step process involving high-temperature pyrolysis of melamine followed by thermal annealing at 200 °C in N_2_ to reinforce interfacial coupling among the components. In this architecture, g-C_3_N_4_, a nitrogen-rich 2D material, served as an effective dispersing matrix for Co_3_O_4_ nanoparticles, preventing their agglomeration and thereby increasing the exposure of active catalytic sites. The resulting ternary catalyst achieved an overpotential of 247 mV at 10 mA cm^−2^, significantly lower than that of pristine Ti_3_C_2_T*_x_* MXene (600 mV) and a Co_3_O_4_/g-C_3_N_4_ binary system (387 mV) (Figure 10e,f), with only small positive shifts of the LSV curve after 1000 CV cycles. This enhancement was attributed to the synergistic electronic interactions among Co_3_O_4_, g-C_3_N_4_, and Ti_3_C_2_T*_x_*, which facilitated efficient adsorption of oxygen intermediates and reduced the OER activation energy.

**Figure 11:**
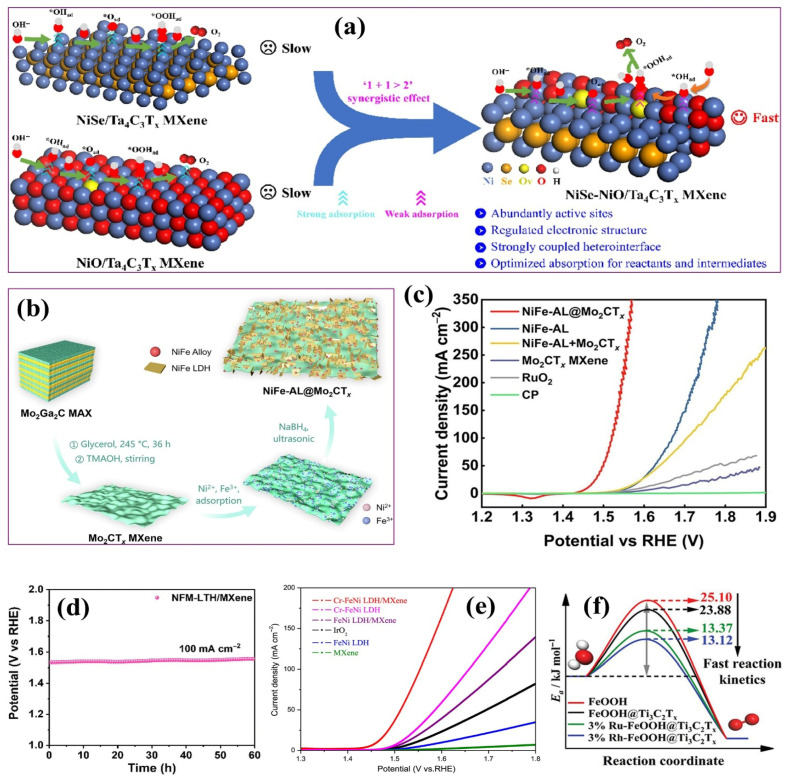
(**a**) Proposed OER mechanism for NiSe/Ta_4_C_3_T*_x_* MXene, NiO/Ta_4_C_3_T*_x_* MXene, and NiSe–NiO/Ta_4_C_3_T*_x_* MXene electrocatalysts. Reproduced with permission from [133]. (**b**) Schematic representation of the synthesis process of NiFe-AL@Mo_2_CT*_x_* MXene and (**c**) LSV curves for NiFe-AL@Mo_2_CT*_x_*, NiFe-AL, NiFe-AL+ Mo_2_CT*_x_*, Mo_2_CT*_x_* MXene, RuO_2_, and carbon paper (CP) at 5.0 mV s^−1^. Reproduced with permission from [136]. (**d**) Stability test of NFM-LTH/MXene at 100 mA cm^−2^ using 1.0 M KOH. Reproduced with permission from [137]. (**e**) LSV curves for MXene, IrO_2_, FeNi LDH, FeNi LDH/MXene, Cr-FeNi LDH, and Cr-FeNi LDH/MXene. Reproduced with permission from [92]. (**f**) E_a_ values of FeOOH, FeOOH@Ti_3_C_2_T*_x_* MXene, 3%Ru-FeOOH@Ti_3_C_2_T*_x_* MXene, and 3%Rh-FeOOH@Ti_3_C_2_T*_x_* MXene electrocatalysts. Reproduced with permission from [138].

In another notable study, Yan et al. [133] developed a NiSe/NiO/Ta_4_C_3_T*_x_* MXene nanohybrid synthesized via a selenization-assisted route, resulting in a hierarchical architecture enriched with heterojunction interfaces. NiSe imparted high electrical conductivity and redox flexibility within this composite, while NiO provided abundant oxygen-containing active sites critical for OER catalysis. The optimized composite delivered an overpotential of 255 mV and a Tafel slope of 47.7 mV dec^−1^. Moreover, it demonstrated excellent electrochemical stability, maintaining consistent activity over 50 h of CP testing without degradation. The improved electrocatalytic behavior was ascribed to the synergistic integration of the highly conductive MXene scaffold, which enabled rapid electron transport and oxygen vacancies in the composite. These vacancies facilitated favorable adsorption and desorption kinetics of key OER intermediates, accelerating the reaction pathway (Figure 11a).

#### 4.2.2. LDH–MXene Composites

LDHs have attracted substantial attention as OER electrocatalysts [134] due to their compositional tunability, rich redox chemistry, and intrinsically high surface area. Their layered structure allows flexible modulation of divalent and trivalent metal cation M^2+^/M^3+^ ratios (e.g., Ni^2+^, Co^2+^, Fe^3+^), enabling precise control over electronic configuration and catalytic behavior. However, pristine LDHs typically suffer from poor electrical conductivity, structural instability, and nanosheet agglomeration [135], which limit catalytic efficiency, especially at high current densities. Researchers have designed LDH–MXene composites to overcome these drawbacks, wherein MXenes serve as highly conductive, chemically stable, and mechanically robust 2D supports. The MXene scaffold enhances charge transport, prevents agglomeration of LDH nanosheets, and preserves catalyst integrity under oxidative conditions, resulting in improved catalytic kinetics and durability. For example, Yang et al. [136] synthesized a NiFe alloy/LDH hybrid anchored on Mo_2_CT*_x_* MXene (NiFeOOH@Mo_2_CO_2_) via a room-temperature liquid-phase reduction route (Figure 11b). The hybrid system achieved an overpotential of 230 mV at 10 mA cm^−2^ in 1.0 M KOH electrolytes (Figure 11c). It exhibited excellent stability over 3000 CV cycles, with the LSV curves showing only a slight shift. In addition, post-analysis revealed that the catalyst retained its original morphology, with NiFe species remaining firmly anchored on the Mo_2_CT*_x_* surface. The performance enhancement can be attributed to the synergistic effects of the NiFe alloy and the Mo_2_CT*_x_* MXene. The former modulates the local electronic environment of active sites, while the latter provides a conductive 2D framework that ensures efficient charge transport and mechanical integrity under oxidative conditions. Additionally, the PDOS of the Ni 3d orbitals revealed a downward shift in the d-band center of the Ni atom from −3.082 eV to −3.242 eV due to interfacial electron transfer within the heterojunction. This electronic modulation weakened the adsorption energy of oxygenated intermediates on the NiFeOOH surface, promoting their desorption and improving O_2_ bubble release, enhancing the overall OER kinetics. In a separate study, Pal et al. [137] developed a ternary NiFeMo layered triple hydroxide (LTH) grown on Ti_3_C_2_T*_x_* MXene, forming hierarchical structures with nanoflake morphologies. The resulting heterostructure catalyst delivered overpotentials of 292 mV in alkaline saline water and 340 mV in real seawater at a current density of 100 mA cm^−2^. These results highlight the composite’s excellent ion diffusion, abundant exposure of active sites, and high durability (over 60 h CP at 100 mA cm^−2^), as shown in Figure 11d. In another research, Yan et al. [92] developed a Cr-doped FeNi-LDH vertically grown on Ti_3_C_2_T*_x_* MXene via an in situ synthesis strategy. The Cr incorporation optimized the electronic structure and created abundant catalytic centers, while the conductive MXene matrix ensured efficient charge transfer. The composite achieved an overpotential of 232 mV and a Tafel slope of 54.4 mV dec^−1^ (Figure 11e). Its efficiency significantly outperformed that of the pristine FeNi-LDH (372 mV), FeNi-LDH/MXene (294 mV), and Cr-doped FeNi-LDH (280 mV), confirming the synergy of dopant engineering and interfacial conductivity. Furthermore, Zhang et al. [138] developed a dual-modification strategy by co-doping FeOOH nanoarrays with Ru and Rh cations and introducing oxygen vacancies. The arrays were grown in situ on Ti_3_C_2_T*_x_* MXene, which enhanced structural integrity and conductivity. The resulting catalyst exhibited a low overpotential of 223 mV and a Tafel slope of 63.6 mV dec^−1^, along with a significantly enhanced turnover frequency. Notably, during the CP test conducted at 10 mA cm^−2^, the Ru- and Rh-doped FeOOH@Ti_3_C_2_T*_x_* catalysts exhibited exceptional stability, maintaining their performance over 100 h without significant decay. The remarkable OER activity stems from the synergistic interplay between cationic doping and oxygen vacancies, which modulate the local electronic structure and optimize the adsorption energies of oxygenated intermediates. After substitutional doping of Fe sites with Ru and Rh, the adjacent Fe–O bond lengths were extended to 1.984 Å and 1.913 Å, respectively, compared to 1.878 Å in pristine FeOOH. This elongation of the Fe–O bonds indicates that Ru or Rh incorporation activates the lattice oxygen, enhances oxygen atom mobility, and facilitates the generation of oxygen vacancies or related defects. Such lattice distortions promote improved charge redistribution and lower the activation energy (E_a_), as shown in Figure 11f, accelerating the OER kinetics and boosting overall catalytic efficiency.

#### 4.2.3. MOF–MXene Composites

**Figure 12:**
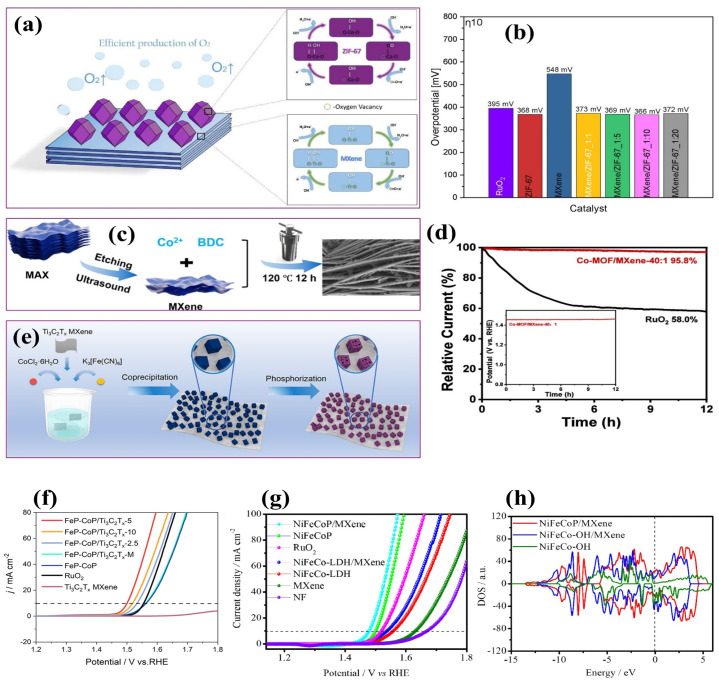
(**a**) Proposed OER mechanism for ZIF-67/Ti_3_C_2_T*_x_* MXene and (**b**) comparison of MXene, RuO_2_, ZIF-67, and ZIF-67/Ti_3_C_2_T*_x_* MXene nanocomposites overpotential (ƞ_10_). Reproduced with permission from [140]. (**c**) Preparation process of Co-MOF/MXene composites with (**d**) its stability tests at controlled potential. Reproduced with permission from [96]. (**e**) Illustration of the preparation of FeP–CoP/Ti_3_C_2_T*_x_* electrocatalyst with (**f**) LSV curves of FeP–CoP/Ti_3_C_2_T*_x_* and control samples. Reproduced with permission from [145]. (**g**) LSV of NF, RuO_2_, NiFeCoP/MXene, NiFeCo-OH/MXene, NiFeCoP, and NiFeCo-OH with (**h**) DOS for NiFeCoP/MXene, NiFeCo-OH/MXene, and NiFeCo-OH. Reproduced with permission from [146].

MOFs, consisting of metal ions/clusters coordinated with organic ligands, have attracted considerable interest as OER electrocatalysis [139] due to their large specific surface area, tunable pore structures, and well-ordered crystalline architectures. These features provide abundant accessible active sites and facilitate efficient diffusion pathways for electrolyte ions and gaseous products. However, pristine MOFs generally suffer from low electrical conductivity and limited long-term electrochemical stability, hindering their practical application in water oxidation. To tackle these challenges, MOFs have been integrated with highly conductive substrates, such as MXenes, leveraging the complementary advantages of both materials. MXenes offer excellent electrical conductivity, hydrophilicity, and structural versatility, promoting rapid charge transport and mechanical stability in composite catalysts. Sroda et al. [140] fabricated a hybrid electrocatalyst by anchoring a zeolitic imidazolate framework (ZIF-67) onto Ti_3_C_2_T*_x_* MXene nanosheets (Figure 12a). The resulting ZIF-67/Ti_3_C_2_T*_x_* composite material exhibited an overpotential of 366 mV at 10 mA cm^−2^ and a Tafel slope of 188.1 mV dec^−1^, significantly outperforming Ti_3_C_2_T*_x_* (548 mV), as shown in Figure 12b. It exhibited enhanced electrochemical stability under CP testing at 20 mA cm^−2^, with only a 0.2% increase in potential after 50 h. The enhanced electrocatalytic activity was attributed to the synergistic interaction between the high-surface-area porous ZIF-67 and the excellent conductivity of Ti_3_C_2_T*_x_* MXene, which not only facilitated rapid charge transfer but also enabled greater accessibility of active sites by improving diffusion across the catalyst surface. In another study, Fang et al. [96] reported the synthesis of a 2D cobalt 1,4-benzenedicarboxylate (Co-MOF) that was uniformly integrated with Ti_3_C_2_T*_x_* via a mild hydrothermal procedure (Figure 12c). This strategy yielded a unique 2D sandwich-like architecture, Co-MOF/MXene, demonstrating excellent OER performance. It requires an overpotential of 390 mV to reach the current density of 100 mA cm^−2^ and retains high catalytic stability over 12 h (95.8%), compared with RuO_2_ (58%), as shown in Figure 12d. The remarkable electrocatalytic behavior stemmed from the intimate interfacial contact and structural compatibility between the Co-MOF and MXene, which enhanced electron mobility and ion accessibility. Notably, the in situ transformation of Co-MOF into Co(OH)_2_ contributed to the activation and durability of the catalytic sites, further stabilizing the OER performance under alkaline conditions.

#### 4.2.4. Metal Phosphide–MXene Composites

Phosphorization has emerged as a pivotal strategy in the development of high-performance OER electrocatalysts [141], owing to its ability to tailor the surface chemistry, electronic structure, and catalytic kinetics of metal-based transition compounds [142]. In particular, the formation of metal phosphides can introduce unique metal-P bonding characteristics that modulate the adsorption energy of key OER intermediates, bringing the ΔG_H*_ closer to the thermoneutral region [143]. This optimizes the reaction pathway and accelerates the overall OER kinetics. Moreover, phosphorization often leads to a porous morphology with an enhanced surface area, improved electrical conductivity, and an increased density of active sites [143,144], all of which are critical for efficient charge and mass transport during electrocatalysis. When integrated with highly conductive and chemically stable substrates, such as MXene, the resultant hybrid structure exhibits synergistic effects that further enhance the electrocatalytic activity and stability under alkaline conditions. A hybrid electrocatalyst comprising FeP-CoP nanostructure anchored on Ti_3_C_2_T*_x_* was synthesized through in situ coprecipitation and a subsequent phosphorization process (Figure 12e) [145]. The composite exhibited a lower overpotential of 270 mV at 10 mA cm^−2^ and a Tafel slope of 49.1 mV dec^−1^ in 1.0 M KOH electrolyte, in comparison to the individual FeP, CoP, and MXene counterparts (Figure 12f). Furthermore, negligible decay was observed after a 20 h CP test, and the LSV curves before and after 5000 CV cycles showed only a minor difference, confirming the excellent electrochemical durability. This enhanced activity was attributed to the synergistic interplay between FeP and CoP, which modulated the adsorption energies of key oxygen intermediates (*OH, *O, *OOH), and the conductive MXene, which provided rapid charge transport pathways and stabilized the nanostructures. In another study, Li et al. [146] synthesized a NiFeCoP/Ti_3_C_2_T*_x_* hybrid catalyst using a two-step strategy involving solvothermal growth followed by post-phosphorization. The resulting hybrid catalyst exhibited excellent OER performance, requiring only an overpotential of 240 mV at 10 mA cm^−2^, a Tafel slope of 55 mV dec^−1^ in 1.0 M KOH solution (Figure 12g), and superior durability. This enhancement is attributed to the synergistic coupling between the trimetallic phosphides and the conductive MXene framework, which modulated the local electronic structure, enriched active sites via heterointerface engineering, and accelerated charge transfer during the OER process, as supported by DFT analyses in Figure 12h. The d-band center of the Ni atom in the NiFeCoP/Ti_3_C_2_T*_x_* system shifted positively from –1.87 eV in NiFeCo–OH to –1.34 eV due to electron withdrawal by the MXene support. This upward shift reduced the occupation of antibonding states between NiFeCoP and the MXene, thereby optimizing the adsorption strength of oxygen-containing intermediates. Such electronic tuning facilitates intermediate desorption and accelerates the overall OER kinetics, ultimately boosting catalytic performance.

Table 4 presents a comparative summary of recent HER and OER studies to provide a clearer perspective on the electrocatalytic performance of MXene-based materials. This table highlights key performance indicators such as overpotential and Tafel slope, offering a quantitative benchmark to evaluate the efficacy of various MXene composites. These comparisons underscore the competitiveness and tunability of MXene-based systems for water-splitting applications.

## 5. Challenges and Future Perspectives

MXenes have emerged as a remarkable category of electrocatalytic materials due to their remarkable electrical conductivity, large specific surface area, and highly tunable surface chemistry. This review has highlighted substantial advances in MXene-based electrocatalysts for HER and OER applications, focusing on their structural versatility, surface functionalization, defect engineering, and heterostructure composites. Despite these promising developments, several significant challenges should be addressed to enable their scalable, durable, and practical application in sustainable energy systems. Conventional MXene synthesis predominantly involves hazardous and corrosive chemical etching agents, such as HF or in situ-generated HF from fluoride salts and hydrochloric acid, posing environmental, health, and safety concerns. Furthermore, these methods lack precise control over MXene surface terminations, resulting in batch-to-batch variability that hampers reproducibility and obscures fundamental understanding of catalytic mechanisms. The current top–down exfoliation approaches also suffer from limited scalability and low throughput. Therefore, developing green, fluorine-free etching protocols, etchant-free exfoliation techniques, or innovative bottom–up synthesis methods is critical. Emerging pathways, such as continuous-flow production, self-assembly, templating, and electrochemical etching, hold promise but require systematic optimization, techno-economic evaluation, and industrial validation.

MXenes are generally composed of earth-abundant elements (e.g., Ti, V, Mo). However, several factors offset their economic advantage, including high energy and chemical costs associated with conventional synthesis routes (e.g., HF or high-temperature molten salt methods). Limitations in scalability lead to increased production cost per unit mass. Surface treatment and delamination steps are often complex and not yet industrialized. However, recent progress in green, scalable synthesis, such as fluorine-free etching and continuous flow processing, has the potential to reduce production costs significantly. Fluorine-free alkali-assisted and electrochemical methods improve safety and lower operational expenses.

One of the most critical challenges confronting MXene-based electrocatalysts is their intrinsic structural and electrochemical instability under operational conditions. These 2D materials are susceptible to degradation pathways, such as restacking and surface oxidation, particularly under harsh electrochemical environments. Such transformations reduce the accessibility of active sites and degrade electronic conductivity, ultimately impairing catalytic performance over time. In HER and OER applications, prolonged exposure to reductive or oxidative potentials can lead to surface reconstruction and the loss of terminal groups (–OH and –F), disrupting active sites and critical reaction pathways. To mitigate these issues, robust protective strategies, such as surface passivation, defect engineering, and rational surface functionalization, are imperative. However, these interventions must carefully balance enhanced stability with preserving MXenes’ intrinsic high conductivity and accessible surface area. Another significant limitation stems from inconsistent control over surface terminations, which play a pivotal role in catalytic activity. Conventional etching techniques, typically employing harsh reagents like HF or LiF–HCl mixtures, lack the precision needed for reproducible and selective tuning of surface groups. This results in batch-to-batch variability and hampers fundamental understanding of how terminations modulate the adsorption energies of key intermediates (*H, *OH, *O, *OOH) in HER and OER. Therefore, advanced synthetic strategies and post-functionalization protocols are essential to achieving selective, uniform, reproducible termination control and optimizing MXene activity and long-term stability. Furthermore, integrating MXenes with complementary materials, such as metal hydroxides, oxides, dichalcogenides, phosphides, and carbon nanostructures, to form heterostructure composites is a promising route to enhance charge transfer, increase active site density, and prevent restacking. Nevertheless, fabricating intimate, coherent, defect-free interfaces that guarantee strong electronic coupling remains technically challenging. Techniques such as ALD, electrostatic assembly, and controlled solvothermal growth offer potential pathways to overcome these interface engineering challenges.

Multi-metal co-doping is an attractive strategy for fine-tuning the local electronic environment and reaction energetics in MXene-based catalysts. By introducing different dopants into the MXene lattice or its hybrid framework, researchers can effectively modulate the d-band center and enhance the adsorption properties toward reaction intermediates. However, multiple dopants’ individual and synergistic influences on the electronic structure and catalytic behavior remain not fully understood. Advanced characterization techniques capable of atomic-scale resolution, such as scanning transmission electron microscopy (STEM) with energy-dispersive X-ray spectroscopy (EDX) and electron energy loss spectroscopy (EELS), alongside DFT simulations, are essential tools for elucidating how structural and compositional complexity impact catalytic performance. A persistent bottleneck in the field is translating laboratory-scale catalytic activity into practical device configurations. Most experimental evaluations are conducted at low catalyst loadings and under idealized electrochemical conditions, which do not fully represent the challenges faced during large-scale deployment. Real-world devices demand electrodes with high areal mass loadings, robust mechanical integrity, optimized porosity to facilitate efficient gas evolution, and minimal internal resistance to sustain high current densities. To address these challenges, researchers are exploring advanced fabrication techniques, including freeze-casting to create hierarchical pore structures, template-assisted assembly to control morphology, and the formulation of printable MXene-based inks to enable scalable manufacturing of high-performance electrodes meeting practical application requirements.

Despite significant progress in developing MXene-based electrocatalysts, reproducible synthesis and scalable manufacturing remain critical challenges for industrial translation. Conventional top–down synthesis routes primarily rely on hazardous etching agents. These methods pose considerable safety and environmental risks while producing MXenes with non-uniform surface terminations and variable interlayer structures, leading to poor batch-to-batch consistency. Such variability undermines reproducibility in electrocatalytic performance and complicates reliable benchmarking across different studies. Furthermore, though effective at the laboratory scale, top–down exfoliation strategies suffer from limited scalability and low product throughput, restricting their commercial viability. The lack of standardized, controllable synthetic protocols impedes the transition toward industrial relevance. Recently, alternative synthetic approaches have gained attention as promising solutions to these obstacles. Fluorine-free or green etching processes aim to reduce hazardous chemical use and improve safety. Continuous-flow production systems offer enhanced scalability and process control, while bottom–up synthesis methods, such as self-assembly or templating techniques, introduce new avenues to engineering well-defined MXene structures with improved uniformity. However, these alternative strategies are still in early development and require systematic optimization. Comprehensive techno-economic analyses and lifecycle assessments are necessary to evaluate their feasibility for large-scale commercial production. Advancing these approaches will be crucial for realizing the full potential of MXene-based electrocatalysts in sustainable, scalable hydrogen generation technologies.

The community urgently needs standardized performance evaluation protocols and deeper mechanistic understanding to accelerate the transition of MXene-based electrocatalysts from laboratory discoveries to practical applications. Discrepancies in experimental methodologies, such as variations in testing protocols, choice of reference electrodes, electrolyte compositions, and reporting formats, pose significant barriers to meaningful cross-comparison and benchmarking across studies. Key catalytic parameters, including TOF, Faradaic efficiency, E_CSA_, and long-term operational stability, are frequently omitted or inconsistently measured, further complicating the assessment of intrinsic catalyst performance. Addressing these challenges requires adopting universally accepted standard testing protocols that reflect realistic operating conditions. Complementing these protocols with advanced in situ and operando characterization techniques is essential to unravel the dynamic structural and chemical changes during catalysis. Techniques such as in situ XRD, Fourier-transform infrared spectroscopy (FT-IR), Raman spectroscopy, and differential electrochemical mass spectrometry (DEMS) provide valuable information on phase evolution, intermediate species, and reaction pathways. Moreover, state-of-the-art operando methods, including TEM and ambient-pressure XPS, can elucidate structure–property relationships at atomic and molecular scales under reactive environments. On the computational side, high-throughput DFT screening combined with machine learning algorithms offers a powerful approach to rapidly identify promising MXene compositions and heterostructures with tailored electronic structures and optimized adsorption energies for reaction intermediates. Such data-driven strategies can guide experimental efforts towards rational catalyst design with improved activity, selectivity, and stability. Ultimately, these multidisciplinary advances, spanning material design, synthesis control, interface engineering, mechanistic exploration, and device integration, will underpin the successful incorporation of MXene-based electrocatalysts into comprehensive water-splitting systems. Coupling these catalysts with photoelectrochemical (PEC) and electrochemical assemblies powered by renewable energy sources will enable flexible, on-demand hydrogen production, aligning with global efforts towards a sustainable, carbon-neutral hydrogen economy.

**Figure 13:**
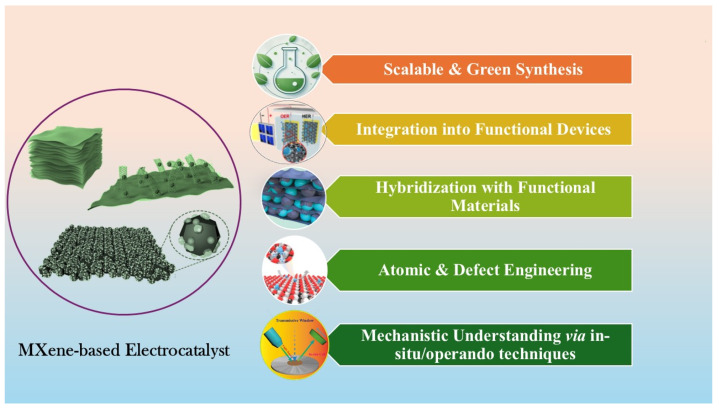
Schematic illustration outlining future perspectives and strategic directions for developing MXene-based materials in electrocatalytic water-splitting applications.

A complex interplay exists among structural configuration, surface chemistry, and interfacial interactions, dictating the electrocatalytic performance of MXene-derived materials. Innovative design strategies, such as precise tailoring of surface features, incorporation of active materials, and creation of multifunctional interfaces, are essential for optimizing critical factors, including adhesion, charge transport, and material durability. By harnessing these synergistic effects, researchers can systematically enhance the intrinsic activity and long-term stability of MXene-based electrocatalysts. Such holistic, interface-focused approaches will be pivotal in bridging laboratory-scale discoveries with the practical deployment of efficient, scalable, and sustainable hydrogen production systems. As the global energy landscape advances toward carbon neutrality, these innovations will be a foundation for next-generation water-splitting technologies and integrated renewable energy platforms. The key points and future directions of MXene-based materials are illustrated in Figure 13.

## Figures and Tables

**Table 1:** Electrocatalytic performance metrics and MXene-specific design strategies.

Performance Metric	Definition/ Significance	Evaluation Method	MXene-Specific Considerations
Overpotential	Additional potential required beyond the thermodynamic value to drive HER/OER	Linear sweep voltammetry (LSV)	Reduced via heteroatom doping, interlayer spacing, and hybridization
Tafel Slope	Indicates reaction kinetics and charge transfer efficiency	Tafel analysis from LSV	Improved by structural tuning, defects, and atomic-scale doping
Exchange Current Density (j_o_)	Intrinsic rate of electron transfer at the equilibrium potential	Extrapolated from Tafel plots	Sensitive to surface termination; enhanced with conductive additives
Turnover Frequency (TOF)	Reactant molecules converted per active site per unit time	Estimated via E_CSA_	Depending on accurate site quantification, reflects intrinsic catalytic activity
Durability/Stability	Catalyst’s ability to retain performance over time	Chronoamperometry, chronopotentiometry, and cycling voltammetry	MXenes show good stability due to a robust structure and modifiable surfaces

**Table 2:** Diagnostic techniques for MXene-based electrocatalysts in HER/OER studies.

Technique	Information Provided	Application in Electrocatalysis	MXene-Specific Insights
TEM/HR-TEM	Morphology, particle size, layer thickness, lattice fringes	Reveals nanoscale structural features, defects, and stacking	Identifies exfoliation quality, interlayer spacing, and hybrid structures
XPS	Surface elemental composition, oxidation states, bonding environments	Tracks surface chemistry evolution, oxidation, and doping effects	Confirms terminations (–O, –F, –OH), dopant incorporation, and post-reaction changes
Raman Spectroscopy	Vibrational modes of chemical bonds and structural defects	Detects phase changes, disorder, and oxidation	Monitors degradation and structural disorder
XRD	Crystalline structure, interlayer spacing	Detects structural changes pre- and post-reaction	Tracks restacking, interlayer expansion, and hybrid phase formation
EIS	Charge transfer resistance, ion diffusion, capacitance	Evaluates charge transport kinetics	Assesses conductivity enhancements via doping/composites
CV Cycling	Reversibility and stability over cycles	Used for long-term durability tests	Tracks degradation in HER/OER cycles
CA CP	Current or potential retention over time	Tests for long-term operational durability	Quantifies time-dependent stability under HER/OER

**Table 3:** Quantitative comparison of HER performance between pristine MXenes and engineered MXene-based composites using ΔG_H*_ values and HER overpotentials (η_10_).

Catalyst	Gibbs Free Energy (ΔG_H*_, eV)	Overpotential (ƞ_10_, mV)	[Ref.]
Ti_3_CNT*_x_* MXene	+0.8	329	[106]
Pt/Ti_3_CNT*_x_* MXene	–0.11	28	
V_2_CT*_x_* MXene	+1.23	450.8	[107]
NiPt/V_2_CT*_x_* MXene	−0.67	11.9	
Ru/rGO	−0.36	70	[111]
Ru/Ti_3_C_2_T*_x_* MXene/rGO	−0.18	42	
RuSA + RuNP-N/C	~−0.65	42	[112]
RuSA + RuNP-N/C-MXene	~0.00	17	
Ti_3_C_2_T*_x_* MXene	−0.312	155	[114]
CoSnO_3_/Ti_3_C_2_T*_x_* MXene	+0.015	45	
Ti_3_C_2_T*_x_* MXene	−0.18	449	[121]
CoP/Ti_3_C_2_T*_x_* MXene	−0.03	135	

**Table 4:** Performance comparison of MXene-based composites for HER and OER.

Catalyst	Application	Electrolyte	Current Density (mA cm^−2^)	Overpotential (mV)	Tafel Slope (mV dec^−1^)	[Ref.]
CoMoSe_2_@ Ti_3_C_2_T*_x_*	HER	1.0 M KOH	10	82	124	[147]
Co-ReS_2_@Ti_3_C_2_T*_x_*	HER	1.0 M KOH	10	65	92	[148]
CoNi(OH)_2_@ Ti_3_C_2_T*_x_*	HER	1.0 M KOH	10	73	85	[149]
PtNi-NiO*_x_*/Ti_3_C_2_T*_x_*	HER	1.0 M KOH	10	24	56.4	[150]
Fe_2_B/Ti_3_C_2_T*_x_*	HER	1.0 M KOH	100	294	92.0	[151]
Ni_1.5_Co_1.5_(PO_4_)_2_@Ti_3_C_2_T*_x_*	OER	1.0 M KOH	100	286	84.0	[47]
NiFeMo/Ti_3_C_2_T*_x_*	OER	1.0 M KOH	100	280	56.0	[137]
CoP@C/Ti_3_C_2_T*_x_*	OER	1.0 M KOH	10	235	54.0	[152]
NiCoSe/Ti_3_C_2_T*_x_*	OER	1.0 M KOH	10	170	66.7	[153]
CeCoFePLDH@ Ti_3_C_2_T*_x_*	OER	1.0 M KOH	10	266	35.1	[154]

## Abbreviations

The following abbreviations are used in this manuscript:

OER	Oxygen evolution reaction
HER	Hydrogen evolution reaction
RHE	Reversible hydrogen electrode
MOFs	Metal–organic frameworks
2D	Two-dimensional
LDHs	Layered double hydroxides
ΔG_H*_	Gibbs free energy of hydrogen adsorption
η	Overpotential
j_o_	Exchange current density
TOF	Turnover frequency
E_CSA_	Electrochemically active surface area
CA	Chronoamperometry
CP	Chronopotentiometry
CV	Cyclic voltammetry
XRD	X-ray diffraction
XPS	X-ray photoelectron spectroscopy
TEM	Transmission electron microscopy
EIS	Electrochemical impedance spectroscopy
HF	Hydrofluoric acid
DES	Deep eutectic solvents
TMAOH	Tetramethylammonium hydroxide
TMDs	Transition metal dichalcogenides
GQDs	Graphene quantum dots
TDOS	Total density of states
DFT	Density functional theory
LTH	Layered triple hydroxide
Ea	Activation energy
ZIF-67	Zeolitic imidazolate framework
STEM	Scanning transmission electron microscopy
EDX	Energy-dispersive X-ray spectroscopy
EELS	Electron energy loss spectroscopy
FT-IR	Fourier-transform infrared spectroscopy
DEMS	Differential electrochemical mass spectrometry
PEC	Photoelectrochemical
